# Multifunctional Cu_2-x_Se nanomaterials for cancer theranostics: recent advances, therapeutic mechanisms, and translational challenges

**DOI:** 10.3389/fchem.2026.1902573

**Published:** 2026-09-22

**Authors:** Jingchao He, Jiachao Xie, Yunxiao Peng, Haicheng Yu, Lina Chen, Xiaohong Wang, Haibo Sun

**Affiliations:** 1 School of Basic Medical Sciences and School of Public Health, Faculty of Medicine, Yangzhou University, Yangzhou, China; 2 Key Laboratory of the Jiangsu Higher Education Institutions for Nucleic Acid and Cell Fate Regulation (Yangzhou University), Yangzhou, China; 3 Department of Medical Oncology and Hematology, Keerqin District First People’s Hospital, Tongliao, China

**Keywords:** Cu_2-x_Se, diagnosis, multifunctional nanomaterial, therapy, tumor

## Abstract

Cu_2-x_Se has made many breakthroughs in tumor therapy due to its unique advantages. Cu_2-x_Se can enhance immunotherapy by multiple mechanisms, such as reshaping the immunosuppressive tumor microenvironment (TME) and triggering immunogenic cell death. Cu_2-x_Se possesses a variety of nanozyme-like activities that can disrupt the redox homeostasis of tumor cells and modulate the TME, enabling chemodynamic therapy (CDT) and improving the efficacy of multiple therapeutic modalities. Cu_2-x_Se not only acts as a cuproptosis inducer and an autophagy inhibitor, but also induces cell-cycle arrest and impairs mitochondrial function, thereby suppressing tumor proliferation and metastasis. Cu_2-x_Se is a multifunctional nanotheranostic agent that not only possesses photoacoustic imaging (PAI) property, but also exhibits excellent thermoelectric catalytic therapy (TECT), photothermal therapy (PTT) and photodynamic therapy (PDT) functions, making it suitable for combined therapy and imaging-guided therapy. At present, studies on Cu_2-x_Se-based tumor therapy have reported a series of encouraging findings that warrant systematic summary and analysis to promote the development of nanomedicine. So far, there is no review article on the applications of Cu_2-x_Se in tumor diagnosis and therapy. In this review, we summarize the recent progress and prospects of Cu_2-x_Se in cancer diagnosis and therapy, focusing on the breakthroughs of Cu_2-x_Se in new strategies for tumor diagnosis and treatment and in solving medical problems. This review intends to offer insights that may accelerate the development of Cu_2-x_Se-based nanomedicine for cancer therapy.

## 1 Introduction

Nanomedicine enables integrated cancer diagnosis and therapy, thereby improving diagnostic accuracy and therapeutic efficacy (Das, 2025; Tong et al., 2025; Zhang et al., 2024b). Nanomedicine-based technologies facilitate the resolution of challenges in cancer treatment and represent a Frontier for developing novel, safe and effective strategies for cancer diagnosis and therapy (Huang et al., 2025; Jia et al., 2025; Jiang and Lin, 2025; Lan et al., 2024; Ma et al., 2025). Several nanomaterials have already entered clinical practice for cancer diagnosis and therapy, demonstrating promising antitumor efficacy and prolonging survival in patients with cancer (Wang Y. et al., 2024; Yin et al., 2024). Safe and effective nanomaterials are fundamental to successful nanomedicine-based cancer therapy (Cao et al., 2024; Liu et al., 2022, Liu J. et al., 2023). Systematically identifying and reviewing highly effective and safe nanomaterials can provide valuable insights into advancing innovative strategies for tumor theranostics (Bindra et al., 2023; Li B. et al., 2024; Wang A. et al., 2024, Wang P. et al., 2024). Copper-based nanomedicines can induce tumor cell death through various death mechanisms, such as pyroptosis, ferroptosis, cuproptosis and apoptosis, making them a promising nanoplatform for the development of novel tumor treatment strategies (Xia et al., 2025). Copper-based nanomedicines can improve the efficiency of photodynamic therapy (PDT), lead to effective photothermal therapy (PTT) and sonodynamic therapy (SDT), and enhance the efficiency of immunotherapy, demonstrating enormous research value (Xia et al., 2025). As a copper-based nanomedicine, Cu_2-x_Se displays significant advantages in cancer therapy and has yielded numerous encouraging results in cancer monotherapy and combination therapy, demonstrating the qualities of a “star” nanomaterial (Chan et al., 2023; Chen et al., 2025; Cheng H. et al., 2025; Lin et al., 2025; Shen et al., 2025; Shi L. et al., 2025; Wang T. et al., 2024; Yang et al., 2024; Yao et al., 2025; Yin et al., 2025).

The advantages of Cu_2-x_Se in tumor diagnosis and treatment primarily include the following aspects. (1) Cu_2-x_Se significantly enhances immunotherapy through multiple mechanisms. Cu_2-x_Se can lead to immunogenic cell death (ICD) and promote release of tumor-associated antigens (Wang T. et al., 2023); Cu_2-x_Se suppresses the expression of the metabolic immune checkpoint 5′-nucleotidase (CD73) and reverses the immunosuppressive state (Zhang H. et al., 2024); Cu_2-x_Se activates the ROS-TRAF6-IRF5-IL-23 signaling pathway, causing tumor-associated macrophages to repolarize toward the tumor-suppressive M1 macrophages and remodel the immunosuppressive microenvironment (Zheng et al., 2022); Cu_2-x_Se can serve as an effective nanocarrier for immunotherapeutic agents (Wang T. et al., 2023). (2) Cu_2-x_Se modulates the TME through various nanoenzyme activities, thereby disrupting cellular redox homeostasis and contributing to tumor therapy. Because it possesses intrinsic peroxidase (POD)-like and catalase (CAT)-like catalytic activities, Cu_2-x_Se can catalyze the high levels of intracellular hydrogen peroxide (H_2_O_2_) to generate reactive oxygen species (ROS) and oxygen (O_2_), thereby enabling chemodynamic therapy (CDT) and enhancing the efficacy of O_2_-dependent treatment modalities including radiation therapy (RT) and PDT (Chen X. et al., 2022; Hu Y. et al., 2022; Liang et al., 2024); Cu_2-x_Se exhibits glutathione oxidase (GSHox) and nicotinamide adenine dinucleotide (NADH) oxidase-like (NOX) activities, which can deplete intracellular glutathione (GSH) and NADH, the major antioxidant molecules, thereby reducing ROS scavenging, disrupting the redox homeostasis, and enhancing the efficacy of ROS-dependent therapies such as CDT, RT, and PDT (Chen X. et al., 2022; Hu Y. et al., 2022; Liang et al., 2024). (3) Cu_2-x_Se not only acts as a cuproptosis inducer and an autophagy inhibitor, but also suppresses mitochondrial respiration and impairs energy metabolism, thereby directly inhibiting tumor metastasis and proliferation (Chan et al., 2023; Liu et al., 2019, Liu Y. et al., 2023). (4) Cu_2-x_Se is a highly efficient multifunctional diagnostic and therapeutic agent that not only exhibits near-infrared II (NIR-II) photoacoustic imaging (PAI) capabilities but also demonstrates NIR-II PTT, PDT, and thermoelectrocatalytic therapy properties, making it highly suitable for cancer theranostics (Pan et al., 2024; Yang et al., 2024; Zhang et al., 2019). (5) Cu_2-x_Se exhibits good biosafety (Luan et al., 2022; Yang et al., 2024). Since both copper and selenium are essential trace elements in the human body, Cu_2-x_Se demonstrates good biocompatibility (Yang et al., 2024); Cu_2-x_Se can be easily synthesized into ultrafine nanoparticles and excreted via the kidneys, thereby reducing the cumulative toxicity of the nanomaterial (Luan et al., 2022). Given these advantages, Cu_2-x_Se has enabled several novel therapeutic and diagnostic strategies (Luan et al., 2022; Pan et al., 2024; Zhang H. et al., 2024). Meanwhile, Cu_2-x_Se has shown significant potential in addressing medical challenges such as radiation resistance (Jiang W. et al., 2024), chemotherapy resistance (Huang et al., 2021), the low efficacy of catalytic therapy caused by high GSH expression and insufficient endogenous H_2_O_2_ (Hu Y. et al., 2022), and the limited efficacy of immunotherapy resulting from an immunosuppressive microenvironment (Zheng et al., 2022). Therefore, Cu_2-x_Se holds great research significance in tumor theranostics. Therefore, it is necessary to comprehensively review the research progress on Cu_2-x_Se in this field, thereby facilitating in-depth exploration of Cu_2-x_Se to promote the development of cancer therapy.

To date, there are no review articles regarding the application of Cu_2-x_Se in tumor diagnosis and therapy. This review timely and systematically summarizes the research progress regarding Cu_2-x_Se in tumor theranostics (Figure 1), and analyzes the challenges and future prospects of its clinical translation. This review focuses on recent advances in the application of Cu_2-x_Se for overcoming challenges in tumor diagnosis and treatment, aiming to provide insights into overcoming bottlenecks in cancer therapy and promoting effective advancements in this field.

**FIGURE 1:**
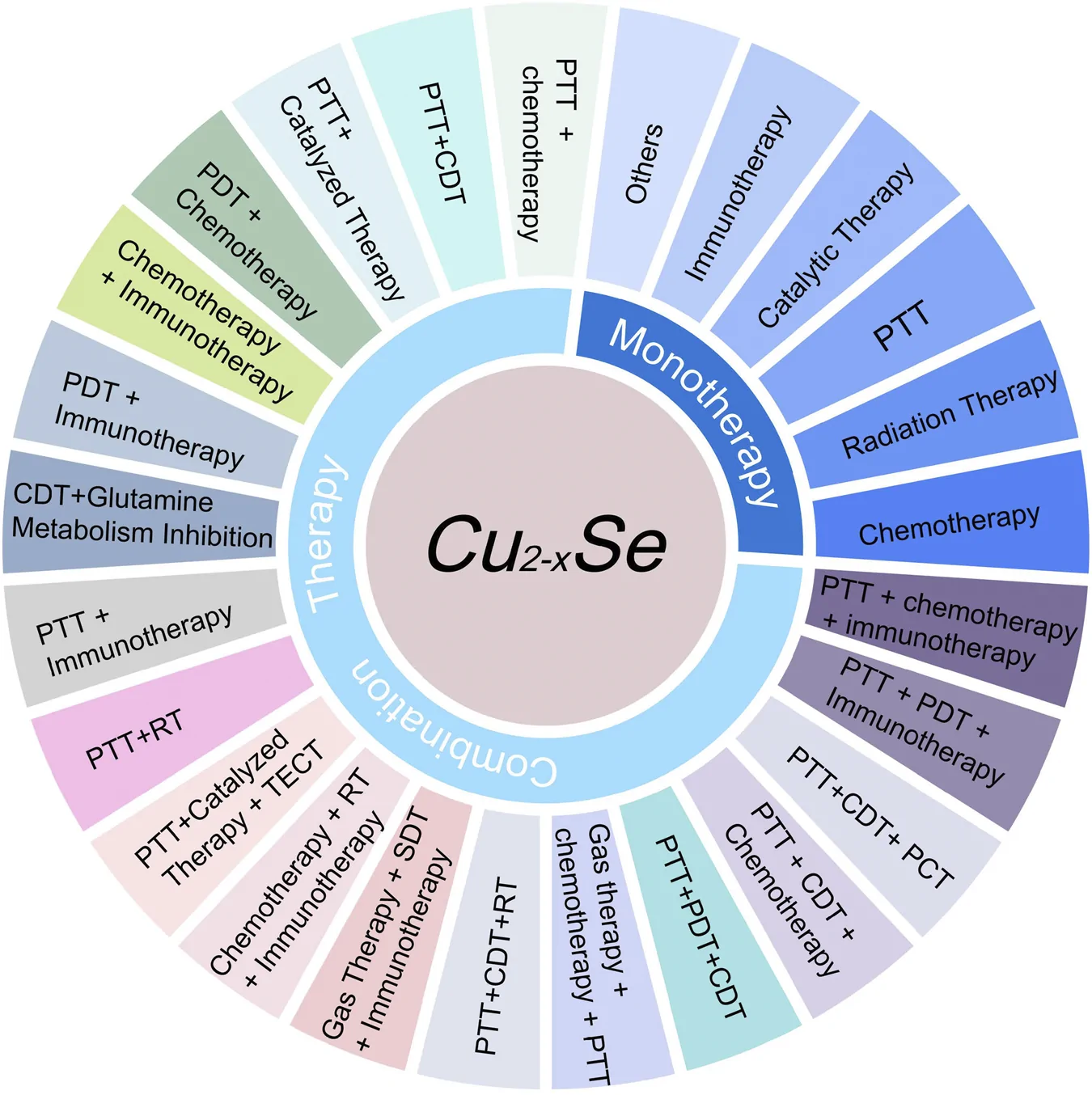
Schematic illustration of the application of Cu_2-x_Se in tumor therapy.

## 2 Applications of Cu_2-x_Se in cancer diagnosis and treatment

### 2.1 Applications of Cu_2-x_Se in monotherapy

Studies on Cu_2-x_Se have reported a series of advances in Cu_2-x_Se-based monotherapeutic applications, including chemotherapy, radiation therapy (RT), PTT, catalytic therapy, and immunotherapy, thereby promoting the development of nanomedicine-based cancer therapy. Recent progress in Cu_2-x_Se-based monotherapy is summarized in Table 1.

**TABLE 1:** Applications of Cu_2-x_Se in monotherapy.

Cu_2-x_Se-based nanosystem	Surface modification	Imaging	Treatment options	PCE	Tumor model	Administration route	Refs
Cu_2-x_Se-GOx	-	-	Catalytic therapy	-	Cervical cancer	intravenous injection	Hu Y. et al. (2022)
Cu_2-x_Se	-	-	Autophagy inhibition	-	Hepatocellular carcinoma	intravenous injection	Liu et al. (2019)
Cu_2-x_Se-PEG	PEG	-	CDT	-	Lung cancer	intravenous injection	Wang X. et al. (2022)
DCNV(CSD)	Dendritic cell membrane	-	Immunotherapy	-	Glioblastoma/Melanoma	subcutaneous injection	Wang T. et al. (2024)
CS-J@CM/6	Cancer cell membranes	-	Immunotherapy	-	Glioblastoma	intravenous injection	Wang T. et al. (2023)
Cu_2-x_Se	-	-	Immunotherapy	-	Melanoma	Peritumoral subcutaneous injection	Zheng et al. (2022)
Microneedle patch loaded with Cu_2-x_Se	-	-	Immunotherapy	-	Breast cancer	intravenous injection and intratumoral injection	Wang Z. et al. (2024)
DMMA@Cu_2-x_Se	RGD and DMMA	-	PTT	-	Melanoma	Trans-surgical bed insertion of microneedle patch	Chan et al. (2023)
bio-Cu_2-x_Se	-	-	PTT	76.9%	Breast cancer	intravenous injection	Wang X.-M. et al. (2023)
GPS-CS@PD-1	PD-1	PAI	PTT	54.53%	Breast cancer	intravenous injection	Bao et al. (2022)
Cu_2-x_Se@MnO2	-	T1-MRI	PTT	22%	Colorectal cancer	intravenous injection	Fu et al. (2018)
CCMT	Cancer cell membranes	-	RT	-	Cervical cancer	intravenous injection	Jiang W. et al. (2024)
Au@Cu_2-x_Se	HS-PEG-SH	PAI	RT	-	Glioblastoma	intravenous injection	Xu et al. (2022)
Cu_2-x_Se@PtSe	HS-PEG-SH	PAI	RT	-	Breast cancer	intratumoral injection	Zhou et al. (2020)
Cu_2-x_Se + DSF	-	PAI	Chemotherapy	-	Hepatocellular carcinoma	intravenous injection	Zhang et al. (2022)
DOX-HCu	-	PAI	Chemotherapy	-	Glioblastoma	intravenous injection	Wu et al. (2018)

#### 2.1.1 Chemotherapy

Chemotherapy serves as one of the mainstays of cancer treatment and improves patients’ survival (Tsai et al., 2024). The nonspecific distribution of chemotherapeutic agents often causes toxicity to normal tissues, thereby limiting the clinical application of chemotherapy (Hani et al., 2024; Yassin et al., 2024; Zhao X. et al., 2024). Zhang et al. prepared biodegradable ultrasmall Cu_2-x_Se nanoparticles that released Cu^2+^ in response to the acidic and H_2_O_2_-rich TME, enabling TME-responsive Cu^2+^ release. Zhang et al. administered biodegradable ultrasmall Cu_2-x_Se nanoparticles to tumor-bearing mice via tail vein injection, together with oral administration of disulfiram (DSF), a repurposed FDA-approved drug for alcohol use disorder. This led to the *in situ* assembly of Cu^2+^released in response to the TME and DSF into highly toxic (N,N-diethyldithiocarbamate)-copper (CuET) at the tumor site for precision chemotherapy (Zhang et al., 2022). Cu_2-x_Se catalyzed the conversion of H_2_O_2_ into ROS and O_2_, thereby enhancing the chemotherapeutic efficacy of CuET. This Cu_2-x_Se-based strategy enabled TME-responsive Cu^2+^ release, thereby facilitating the site-specific formation of CuET at the tumor site. This overcame the problem of nonspecific drug distribution, enabling safe and effective tumor-specific chemotherapy. Wu et al. linked ultra-small Cu_2-x_Se particles to disulfide bond-rich hollow mesoporous organosilica nanoparticles (HMONs) through disulfide linkages to prepare HCu. They then loaded doxorubicin (DOX) into the cavities and mesopores of HCu to prepare DOX-HCu (Figure 2A). Because focused ultrasound induced blood–brain barrier (BBB) disruption, DOX-HCu could effectively penetrate the BBB and accumulate in glioblastoma tissues. Because disulfide bonds are highly sensitive to reducing environments, DOX-HCu could undergo degradation (Figures 2B,C) and release DOX in the glutathione (GSH)-rich TME. This design enabled on-demand release of the chemotherapeutic agent in response to the TME, thereby improving the efficacy of chemotherapy and reducing toxic side effects. Ultrasmall Cu_2-x_Se nanoparticles exhibit excellent PAI performance. DOX-HCu enabled PAI-guided chemotherapy and exhibited significant tumor growth inhibition in glioblastoma-bearing mice (Figure 2D) (Wu et al., 2018). This strategy combined biodegradable HMONs with renal-clearable ultrasmall Cu_2-x_Se nanoparticles to form a composite material. This approach may reduce nanomaterial-associated cumulative toxicity and provide a novel nanoscale platform for safe tumor treatment. This study demonstrated that ultrasmall Cu_2-x_Se could be effectively adsorbed onto the surface of larger mesoporous materials through a simple adsorption method to form new composite materials, which simultaneously leveraged the excellent PAI performance of ultrasmall Cu_2-x_Se and overcame its limited drug-loading capacity caused by the lack of active functional groups and mesoporous structures.

**FIGURE 2:**
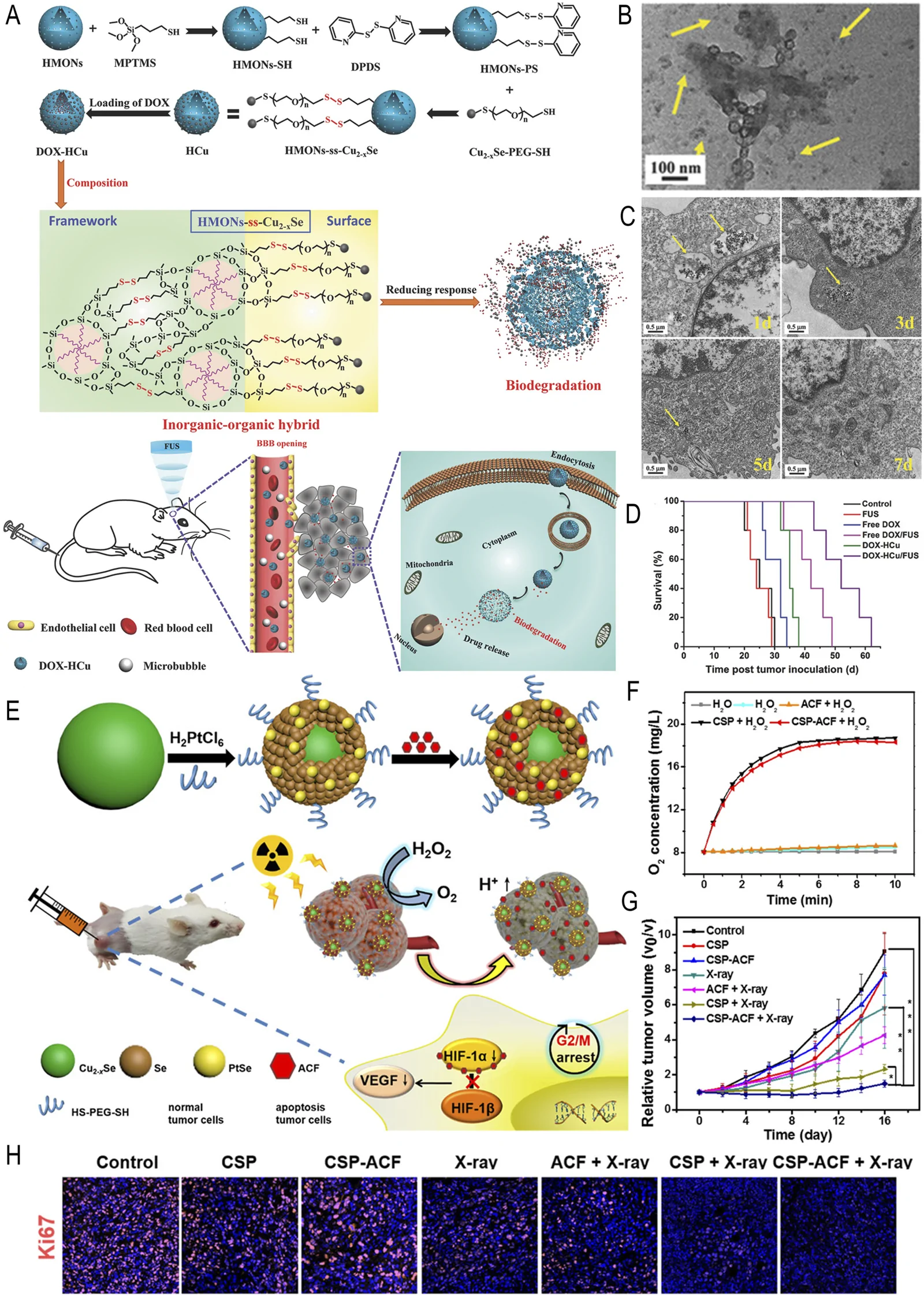
**(A)** Schematic illustration of the preparation process of DOX-HCu and its application in chemotherapy, **(B)** TEM image of HCu in 10 mM GSH solution. **(C)** Bio-TEM images for evaluating degradation ability of HCu in cancer cells. **(D)** Survival times of tumor-bearing mice in different groups. Reproduced from (Wu et al., 2018). Copyright 2018 Advanced Science. **(E)** Schematic illustration of the preparation process of CSP–ACF and its radiosensitization mechanism. **(F)** Evaluation of O_2_ levels in different groups. **(G)** The variation of relative tumor volume in different groups. **(H)** Ki67 staining of tumor tissues in different groups. Reproduced from (Zhou et al., 2020). Copyright 2020 ACS Appl Mater Interfaces.

In summary, Cu_2-x_Se-based nanosystems enabled the site-specific generation of highly toxic chemotherapeutic species at tumor sites and TME-responsive drug release, thereby addressing the limitations of nonspecific chemotherapeutic distribution and unintended drug release in non-tumor areas, which enhanced chemotherapeutic efficacy and reduced adverse effects.

#### 2.1.2 Radiation therapy

RT primarily kills cancer cells by inducing direct DNA damage through ionizing radiation and by generating ROS through reactions with water, which further damages DNA (Zhu et al., 2022). Hypoxia in the TME leads to insufficient ROS production during irradiation, resulting in radiation resistance (Pi et al., 2023; Wang X. et al., 2024; Xiong et al., 2025). Tumor cells often exhibit robust DNA repair capacity, and X-ray irradiation can lead to protective autophagy, both of which induce the development of radioresistance (de Bakker et al., 2024; Porrazzo et al., 2024; Shi et al., 2024). Xu et al. prepared core–shell-structured Au@Cu_2-x_Se (Au@CS) particles with PAI properties. Through multiple radiosensitization mechanisms, these particles achieved highly effective radiotherapy for orthotopic glioblastoma (Xu et al., 2022). Au@CS increased the expression of SQSTM1/p62 and LC3-II proteins to inhibit X-ray-induced protective autophagy, enhancing radiosensitivity through autophagy inhibition. Au@CS increased the ubiquitination of Rad51, a key protein involved in homologous recombination (HR)-mediated DNA damage repair, thereby promoting its proteasomal degradation. This process blocked the critical HR repair pathway and inhibited DNA damage repair, enhancing the efficacy of radiotherapy. Au@CS catalyzed the conversion of H_2_O_2_ into O_2_, improving hypoxia in the TME and increasing tumor radiosensitivity. This study enhanced RT efficacy and mitigated treatment resistance through multiple mechanisms, including alleviation of hypoxia and inhibition of protective autophagy and DNA damage repair, thereby providing new insights into radiosensitization (Xu et al., 2022). There are numerous causes of radiation resistance and multiple pathways for radiosensitization (Krayem et al., 2022). Currently, effective methods for enhancing radiosensitivity include improving hypoxia to increase ROS production during radiotherapy, utilizing high-Z element nanomaterials to enhance the absorption of ionizing radiation, and inducing cell-cycle arrest in the radiosensitive G_2_/M phase (Carigga Gutierrez et al., 2024; Ding et al., 2024; Shestovskaya et al., 2024). Zhou et al. prepared the core–shell-structured Cu_2-x_Se@PtSe (CSP) by utilizing the Kirkendall effect. They then loaded the HIF-1α inhibitor acriflavine (ACF) onto CSP via electrostatic interactions to obtain CSP-ACF (Figure 2E). CSP-ACF contained the high-Z element Pt and catalyzed H_2_O_2_ to generate O_2_, which catalyzed H_2_O_2_ to induce the generation of O_2_, thereby alleviating tumor hypoxia (Figure 2F). CSP-ACF also induced cell-cycle arrest in the radiosensitive G_2_/M phase and suppressed HIF-1α expression, inhibiting tumor cell proliferation. By enhancing radiosensitivity through four complementary mechanisms, CSP-ACF achieved significant radiotherapeutic effects (Figures 2G,H) (Zhou et al., 2020). This study not only provided a multifunctional platform for radiosensitization but also enriched the theoretical framework of radiosensitization research. RT is currently one of the primary clinical tumor treatment modalities, and many patients with cancer require RT (Huang and Zhou, 2020). However, tumors often develop intrinsic or acquired radioresistance, leading to cancer recurrence and metastasis (Darvish et al., 2023). Jiang et al. constructed CCMT by encapsulating Cu_2-x_Se within cancer cell membranes modified with tumor necrosis factor-related apoptosis-inducing ligand (TRAIL). TRAIL specifically binds to death receptors DR4 and DR5 and induces apoptosis in cancer cells. Owing to TRAIL-modified cancer cell membranes, CCMT could evade immune clearance and actively target cervical cancer cells overexpressing DR4/5. Cu_2-x_Se could deplete GSH and catalyze H_2_O_2_ to induce the generation of ROS, disrupting the redox homeostasis of cancer cells and sensitizing them to radiotherapy. Upon irradiation, CCMT induced mitochondrial dysfunction, inhibited oxidative phosphorylation and glycolysis, and blocked energy production, reversing radioresistance. By utilizing different mechanisms, CCMT exhibited highly effective anticancer activity against radioresistant cervical cancer cells (Jiang W. et al., 2024). This study suggested that Cu_2-x_Se can generate ROS and disrupt the redox homeostasis of tumor cells, acting as a radiosensitizer. By employing a combined strategy of disrupting redox homeostasis, inducing mitochondrial dysfunction, and inhibiting energy metabolism, this study reversed radioresistance, thereby expanding the therapeutic strategies for overcoming radioresistance. However, this therapeutic strategy was primarily investigated in cervical cancer cells overexpressing DR4/5, and its ability to reverse radioresistance in cervical cancer cells with low DR4/5 expression was not assessed, limiting the generalizability of this approach.

In summary, the Cu_2-x_Se-based nanosystems can disrupt the redox homeostasis in tumor cells, improve O_2_ levels at the tumor site, inhibit energy metabolism, suppress DNA damage repair, arrest the cell cycle at the G_2_/M phase, and inhibit autophagy, thereby sensitizing tumors to radiotherapy through various mechanisms. Tumor cells are heterogeneous, diverse, and highly adaptive, making them prone to developing resistance to single-mechanism therapies. Importantly, the Cu_2-x_Se-based nanosystems integrated the aforementioned radiosensitization mechanisms into a single platform, synergistically combining these diverse mechanisms to enhance radiotherapeutic efficacy.

#### 2.1.3 PTT

As an effective therapeutic modality, PTT involves photothermal agents absorbing laser energy and converting it into thermal energy, thereby destroying tumors through high temperatures. PTT possesses advantages such as low toxicity, operational simplicity, and high therapeutic selectivity (Delgado-Corrales et al., 2025; Li L. et al., 2024). However, the complicated synthesis of many photothermal agents and their relatively low photothermal conversion efficiencies hinder the development of PTT. Wang et al. added a culture medium containing Cu^2+^ and SeO_3_^2-^ to Shewanella MR-1 to biosynthesize bio-Cu_2-x_Se with high photothermal activity and excellent water solubility through a green biosynthetic approach (Figure 3A). Under 1,064 nm laser irradiation, Bio-Cu_2-x_Se exhibited a photothermal conversion efficiency (PCE) of 76.9% which was significantly higher than that of many reported photothermal agents. Bio-Cu_2-x_Se demonstrated good biosafety and showed highly efficient second near-infrared window (NIR-II) PTT, achieving 87% tumor tissue ablation. This study utilized a simple and environmentally friendly biosynthetic method to prepare photothermal agents with high PCE and good safety, providing new insights into the green synthesis of NIR-II photothermal agents and promoting the development of bacteria-mediated nanocomposite therapeutic platforms (Wang X.-M. et al., 2023). Bao et al. synthesized core–satellite-structured nanoparticles (GPS-CS) by growing gold nanoparticles (AuNPs) on the surface of Cu_2-x_Se. They then prepared GPS-CS@PD-1 by modifying the GPS-CS surface with programmed cell death protein 1 (PD-1). Due to the presence of PD-1, GPS-CS@PD-1 could actively target triple-negative breast cancer cells overexpressing programmed death-ligand 1 (PD-L1), effectively accumulating at the tumor site. Cu_2-x_Se modified with low-toxicity AuNPs could oxidize the more toxic Cu^+^ to the less toxic Cu^2+^, thereby reducing the toxicity of Cu_2-x_Se. GPS-CS exhibited a PCE of 54.53%, which was higher than that of Cu_2-x_Se (35.84%), demonstrating excellent photothermal performance. GPS-CS@PD-1 exhibited excellent biocompatibility, actively targeted triple-negative breast cancer (TNBC), and enabled PAI-guided PTT, markedly suppressing tumor growth and liver metastasis (Bao et al., 2022). This study utilized a simple method to prepare GPS-CS@PD-1 with active targeting ability and high PCE, enabling highly efficient actively targeted PTT for TNBC. Multifunctional photothermal agents with imaging and photothermal properties can enable precise localization of the irradiation region in PTT, thereby improving PTT efficiency and reducing adverse effects (Aboeleneen et al., 2023; Wang R. et al., 2023; Yu et al., 2024). Fu et al. performed a redox reaction of KMnO_4_ onto polyacrylamide-modified Cu_2-x_Se through a simple and mild method to prepare core–shell-structured Cu_2-x_Se@MnO_2_. Cu_2-x_Se@MnO_2_ exhibited a PCE of 22% and enabled T1-weighted MRI-guided NIR-II PTT, effectively inhibiting tumor growth (Fu et al., 2018). This study demonstrated that Cu_2-x_Se was an excellent photothermal agent that could be combined with imaging-capable nanomaterials to construct multifunctional photothermal platforms. Because intracellular copper levels are tightly regulated by copper metabolism and homeostasis, insufficient copper accumulation can lead to inefficient cuproptosis induction (Chan et al., 2023; Chen L. et al., 2022). Developing copper-based nanocarriers capable of site-specifically and efficiently releasing Cu^2+^ at tumor sites can enhance cuproptosis and reduce adverse effects, making it a topic of significant research value (Chan et al., 2023). Chan et al. modified the surface of Cu_2-x_Se with polyethyleneimine (PEI) and the active-targeting agent RGD, and 2,3-dimethylmaleic anhydride (DMMA), resulting in the preparation of DMMA@Cu_2-x_Se. The PEI-mediated positive surface charge markedly enhanced cellular uptake of the nanosystem. DMMA shielded the strongly positively charged PEI on the nanoparticle surface to reduce nonspecific cellular uptake of the nanosystem in the bloodstream. Due to DMMA’s acid-responsive degradation properties, DMMA@Cu_2-x_Se underwent charge reversal in the acidic TME, exposing highly cationic PEI and RGD on the Cu_2-x_Se surface (Figure 3C). Owing to RGD-mediated active targeting and PEI’s strong positive charge, PEI/RGD-exposed Cu_2-x_Se nanoparticles could efficiently enter tumor cells. Researchers used Western blotting (WB) to demonstrate that the Cu^2+^ released by DMMA@Cu_2-x_Se caused oligomerization of dihydrolipoamide S-acetyltransferase (DLAT), downregulated FDX1 protein expression, and caused abnormal expression of tricarboxylic acid (TCA) cycle-related proteins (Figure 3D). These effects inhibited aerobic respiration, induced cuproptosis in tumor cells, and enhanced PTT efficacy. DMMA@Cu_2-x_Se-mediated PTT caused mitochondrial damage and promoted the production of ROS. The generated ROS promoted the release of copper ions, enhanced the cuproptosis effect, and consequently further enhanced the efficacy of PTT. DMMA@Cu_2-x_Se enabled cuproptosis-enhanced PTT, effectively inhibiting tumor growth (Figure 3B) (Chan et al., 2023). DMMA@Cu_2-x_Se served as an effective copper-ion nanocarrier, exhibiting strong copper-ion retention and releasing Cu^2+^ under acidic and H_2_O_2_-rich conditions. This enabled precise and efficient copper-ion release at the tumor site, inducing highly effective cuproptosis and overcoming the issue of low cuproptosis efficacy caused by insufficient copper ions accumulation (Chan et al., 2023).

**FIGURE 3:**
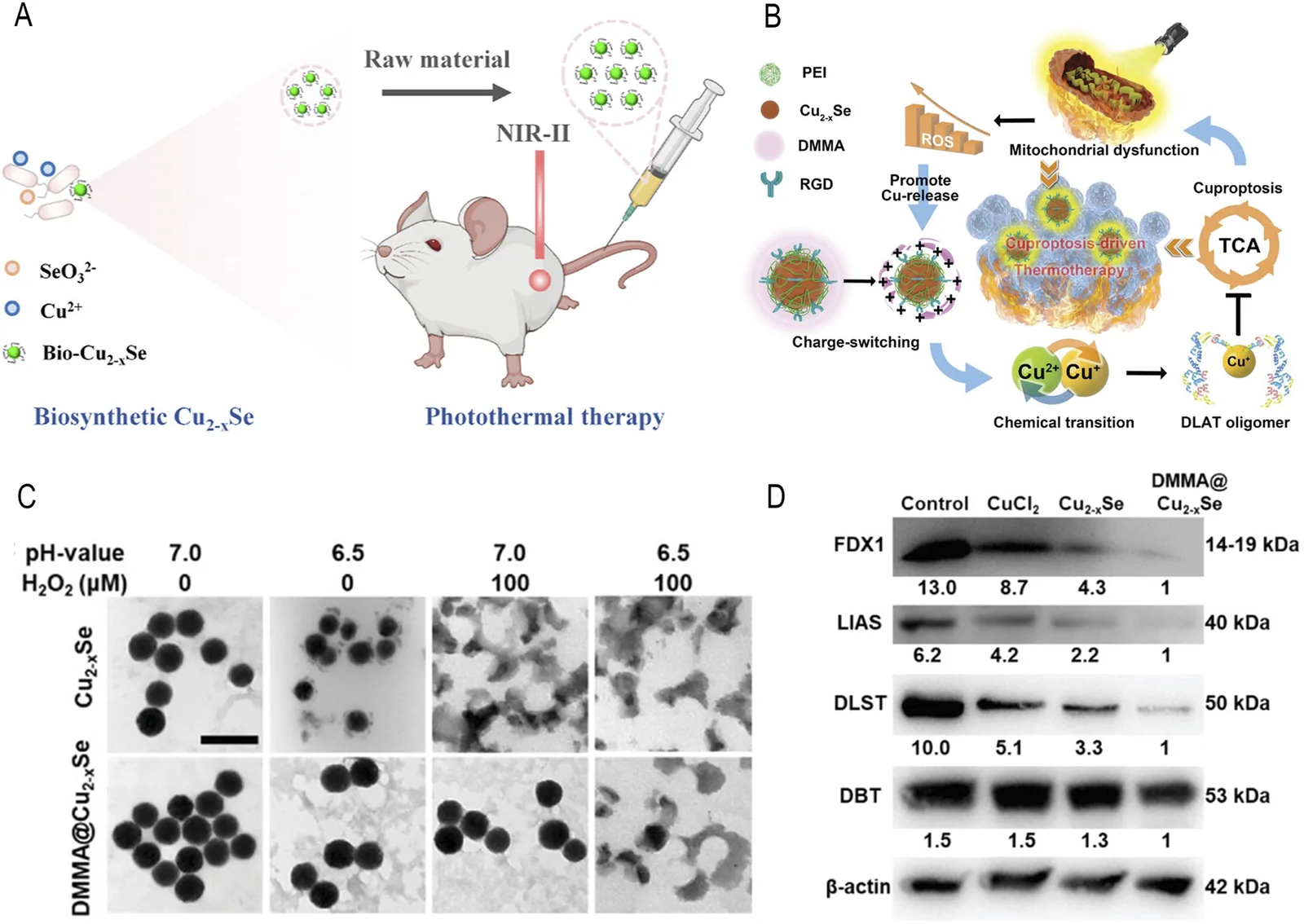
**(A)** Schematic illustration of the preparation process of Cu_2-x_Se and its application in photothermal therapy. Reproduced from (Wang X.-M. et al., 2023). Copyright 2023 ACS Appl Mater Interfaces. **(B)** Schematic illustration of the application of DMMA@Cu_2-x_Se-mediated cuproptosis-enhanced photothermal therapy. **(C)** Degradation behavior of DMMA@Cu_2-x_Se under acidic and H_2_O_2_-rich conditions. **(D)** Changes in the expression of cuproptosis-related proteins regulated by DMMA@Cu_2-x_Se. Reproduced from (Chan et al., 2023). Copyright 2023 Advanced Functional Materials.

In summary, Cu_2-x_Se can enhance PTT efficacy in the following ways: 1) Cu_2-x_Se can be easily synthesized via simple and environmentally friendly methods to produce photothermal agents with high PCE; 2) Cu_2-x_Se can be combined with other photothermal agents to form composites that improve photothermal conversion efficiency; 3) Cu_2-x_Se can be combined with imaging-capable nanomaterials to create multifunctional photothermal agents with simultaneous imaging and photothermal capabilities; 4) Cu_2-x_Se can induce cuproptosis to enhance PTT efficiency; 5) Cu_2-x_Se-based platforms can be modified by active-targeting ligands to effectively accumulate at tumor sites and improve photothermal effects.

#### 2.1.4 Catalytic therapy

Catalytic therapy primarily refers to the application of enzyme-active nanocatalysts to induce catalytic reactions in tumor cells, thereby inducing tumor cell death. One representative catalytic therapy strategy involves converting elevated intratumoral H_2_O_2_ into ROS, thereby leading to tumor cell death (Liu C. et al., 2024). Insufficient intracellular H_2_O_2_ levels and elevated intracellular GSH that acts as an ROS scavenger reduce the efficacy of catalytic therapy (He L. et al., 2023; Li Y. et al., 2023, Li Q. et al., 2024). Hu et al. attached glucose oxidase (GOx) to the surface of NH_2_-PEG-NH_2_-modified Cu_2-x_Se by utilizing amide bonds to obtain Cu_2-x_Se-GOx. Cu_2-x_Se exhibited catalase-like (CAT-like) activity, catalyzing H_2_O_2_ in the TME to generate O_2_ and thereby enabling catalytic reactions activated by the TME. GOx consumed O_2_ to catalyze the conversion of glucose into gluconic acid and substantial amounts of H_2_O_2_, thereby compensating for the deficiency of endogenous H_2_O_2_. Cu_2-x_Se also exhibited peroxidase-like (POD-like) activity, catalyzing the conversion of H_2_O_2_ into hydroxyl radicals (•OH) and thereby killing tumor cells. Cu_2-x_Se also possessed glutathione oxidase-like (GSHOx-like) activity, significantly depleting GSH that scavenged ROS. Cu_2-x_Se-GOx exhibited good biocompatibility and had achieved highly effective catalytic therapy (Hu Y. et al., 2022). By combining nanozymes with biological enzymes, this study demonstrated TME-activated “four-in-one” enzyme-mimicking activities, enabling cascade catalytic reactions. This approach addressed the low efficiency of catalytic therapy induced by elevated intracellular GSH levels and insufficient endogenous H_2_O_2_ in the TME, thereby enabling efficient and safe catalytic therapy (Hu Y. et al., 2022). The TME is characterized by mild acidity, hypoxia, and high levels of GSH and H_2_O_2_, which contributes to radioresistance, chemoresistance, and poor PDT efficacy (Chen et al., 2024a; Liu et al., 2025; Meng et al., 2024). Cu_2-x_Se can exhibit various nanozyme-like activities and reshape the TME by scavenging GSH and catalyzing the decomposition of H_2_O_2_ into O_2_ and •OH. By reshaping the TME, Cu_2-x_Se can enhance the efficacy of multiple cancer therapeutic modalities and show considerable research potential.

#### 2.1.5 Immunotherapy

In some patients, tumor resection may be incomplete, making tumor prone to recurrence. Therefore, developing new strategies to prevent postoperative tumor recurrence is of great value. Microneedle patches are transdermal drug delivery systems consisting of needle arrays with lengths of 25–2000 μm, which enable loaded drugs to achieve high concentrations in the superficial skin layers, rendering them attractive for skin tumor therapy (Ma and Wu, 2017; Wang Z. et al., 2024). Cu_2-x_Se possessed GSHOx and POD activity, which scavenged GSH and catalyzed the conversion of elevated intracellular H_2_O_2_ into ROS, thereby leading to immunogenic cell death (ICD) and enhancing large amounts of tumor-associated antigens (TAAs) release. Wang et al. applied Cu_2-x_Se-loaded microneedle patches (CSN-M) to the postoperative tumor sites in tumor-bearing mice, where they induced ICD and acted as an *in situ* tumor vaccine, resulting in highly effective immunotherapy and significant suppression of postoperative tumor recurrence (Figure 4A) (Wang Z. et al., 2024). This study demonstrated that Cu_2-x_Se can induce ICD in tumor cells when delivered using a simple and cost-effective microneedle-based strategy, making it an effective agent for enhancing the antitumor immune response (Wang Z. et al., 2024). Tumor-associated macrophages are classified into tumor-suppressive M1-like macrophages and tumor-promoting M2-like macrophages (Santarsiero et al., 2024). M1-like macrophages can directly phagocytose and kill tumor cells, effectively present antigens, and activate antitumor T-cell immunity (Wang D. et al., 2024). M2-like macrophages can suppress T cells through recruiting regulatory T cells (Tregs), thereby promoting tumor metastasis and recurrence (DeNardo and Ruffell, 2019; Zheng et al., 2022). Repolarizing M2-like macrophages into M1-like macrophages to remodel the immunosuppressive TME is a key strategy for enhancing immunotherapy (Zheng et al., 2022). Ultrasmall Cu_2-x_Se (CS) particles could activate the ROS-TRAF6-IRF5-IL-23 signaling pathway, ultimately leading to the repolarization of tumor-associated macrophages into M1-like macrophages. This process induced a specific CD8^+^ T-cell immune response and generated a significant immune memory effect in tumor-bearing mice, effectively inhibiting tumor metastasis and growth (Figure 4B) (Zheng et al., 2022). This study demonstrated that CS was an effective immunomodulator with adjuvant-like effects that could remodel the immunosuppressive TME and enhance antitumor immune responses (Zheng et al., 2022). The reduced infiltration and dysfunction of T cells in the tumor region lead to immune evasion, limiting the efficacy of immunotherapy in glioblastoma (Fecci et al., 2014; Wang T. et al., 2023). Wang et al. prepared CS-J@CM/6 b y loading the small-molecule inhibitor JQ1 onto Cu_2-x_Se, coating the prepared nanomaterials with cancer cell membranes, and modifying the surface of the cell membranes with CD6. Wang et al. orally administered paroxetine (PX) and intravenously injected CS-J@CM/6 into tumor-bearing mice. This combination increased infiltration of T-cell in the tumor region and reversed T-cell dysfunction in the TME, yielding significant immunotherapeutic effects (Wang T. et al., 2023). Due to the specific interaction between the activated leukocyte cell adhesion molecule (ALCAM) expressed on endothelial cells and CD6, CS-J@CM/6 could effectively cross the BBB and subsequently actively target cancer cells via homologous targeting. PX acted as a G protein-coupled receptor kinase 2 (GRK2) inhibitor, stabilizing sphingosine-1-phosphate receptor 1 (S1PR1) on the surface of T cells, thereby promoting the release of T cells sequestered in the bone marrow and increasing T-cell infiltration at the tumor site. JQ1 in CS-J@CM/6 not only reduced the expression of immune checkpoint molecules PD-1 and TIM-3 on T cells but also downregulated PD-L1 expression, simultaneously inhibiting multiple immune checkpoints and synergistically enhancing T-cell function and activity. In CS-J@CM/6, Cu_2-x_Se generated ROS via a Fenton-like reaction, thereby inducing ICD in tumor cells and promoting the TAAs release to elicit an immune response. This study increased T-cell infiltration in the tumor region and simultaneously inhibited multiple immune checkpoints to synergistically enhance T-cell function and activity, achieving significant immunotherapeutic effects and providing insights for improving immunotherapy for glioblastoma (Figure 4C) (Wang T. et al., 2023). Dendritic cell (DC)-based vaccines can significantly enhance immunotherapy and demonstrate broad application prospects (Borges et al., 2024; Kumari et al., 2024; Shah et al., 2025). Due to their large size and instability *in vivo*, traditional DC-based vaccines exhibit inefficient lymph-node homing and are unable to efficiently activate cytotoxic T lymphocytes (Park et al., 2006; Wang T. et al., 2024). Insufficient presentation of tumor antigens by DCs and low expression of major histocompatibility complex class I (MHC-I) prevent the efficient activation of CD8^+^ T cells, which are primarily responsible for antitumor immune responses (Saxena et al., 2021; Wang T. et al., 2024). To address these limitations, Wang et al. prepared DCNV(CSD) by coating Cu_2-x_Se with mature DC membranes. CDT mediated by DCNV(CSD) induced ICD in tumor cells, promoting the release of abundant TAAs. Owing to overexpression of MHC-I, the DCNV(CSD) nanovaccine could activate CD8^+^ T cells more effectively. Because of its small size, good stability, and high expression of chemokine receptors CCR7, the DCNV(CSD) nanovaccine could effectively accumulate in lymph nodes, thereby effectively inducing tumor-specific T-cell responses. The DCNV(CSD) nanovaccine induced potent tumor-specific CD8^+^ T-cell responses and generated memory T cells in the spleen of mice, effectively suppressing highly aggressive glioblastoma and highly metastatic melanoma, and effectively preventing tumor recurrence after treatment (Figure 4D) (Wang T. et al., 2024). DCNV(CSD) nanovaccines overcame the limitations of conventional DC-based vaccines, such as low accumulation in lymph nodes and low MHC-I expression, thereby improving immunotherapeutic efficacy (Wang T. et al., 2024).

**FIGURE 4:**
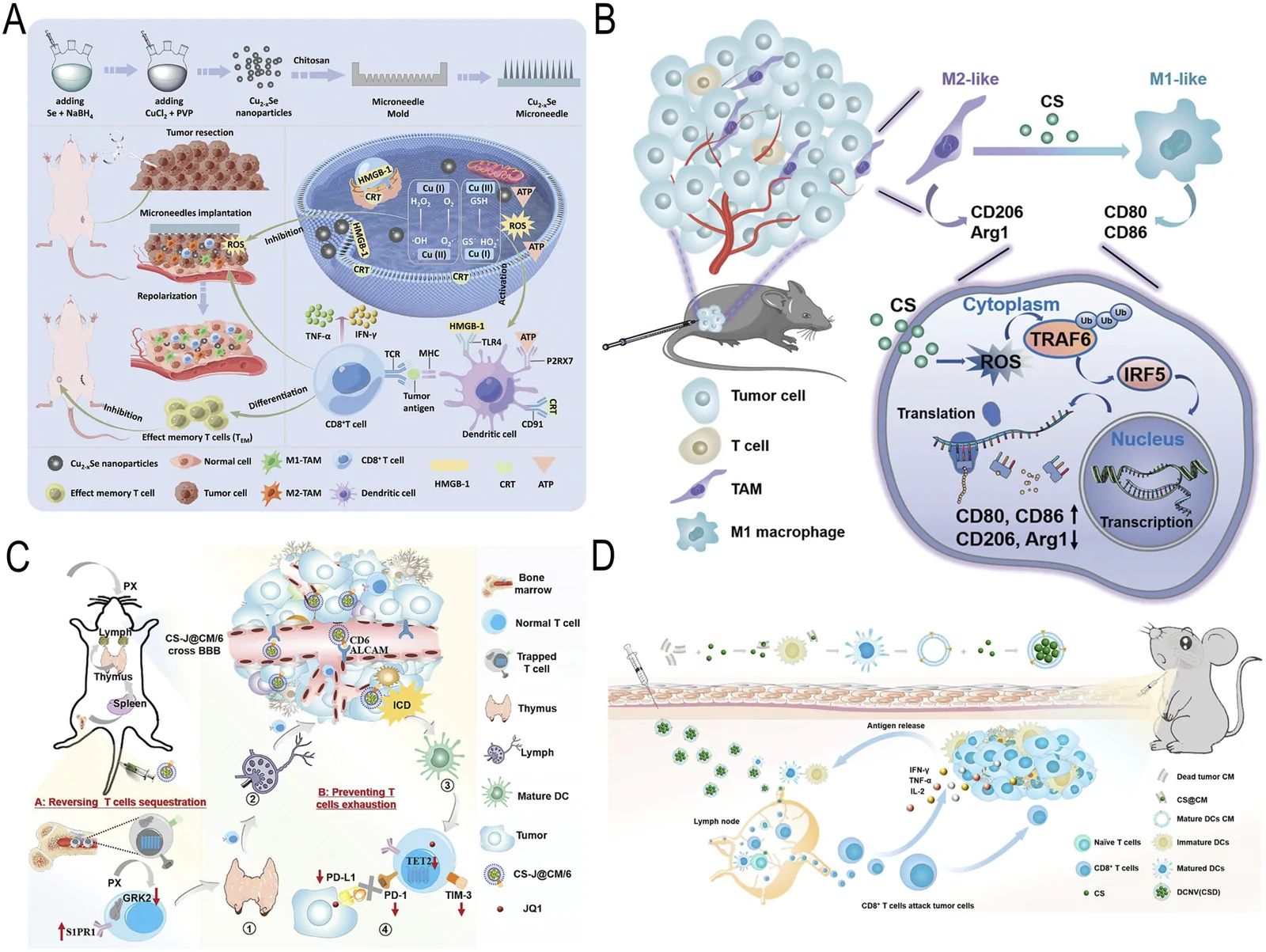
**(A)** Schematic illustration of the preparation process of CSN-M and its antitumor mechanism. Reproduced from (Wang Z. et al., 2024). Copyright 2024 Adv Healthc Mater. **(B)** Schematic illustration of CS-mediated immunotherapy through inducing the repolarization of tumor-associated macrophages to the M1-like phenotype. Reproduced from (Zheng et al., 2022). Copyright 2022 Advanced Functional Materials. **(C)** Schematic illustration of the application of CS-J@CM/6-mediated immunotherapy. Reproduced from (Wang T. et al., 2023). Copyright 2023 Advanced Science. **(D)** Schematic illustration of the synthesis of dendritic cell-based vaccines for highly effective immunotherapy. Reproduced from (Wang T. et al., 2024). Copyright 2024 ACS Nano.

In summary, Cu_2-x_Se can enhance immunotherapy primarily through the following mechanisms: 1) Cu_2-x_Se can induce ICD in tumor cells by generating ROS; 2) Cu_2-x_Se can cause tumor-associated macrophages to repolarize into M1-like macrophages by activating the ROS-TRAF6-IRF5-IL-23 signaling pathway, thereby reshaping the immunosuppressive TME; 3) Cu_2-x_Se can serve as a carrier for immunomodulatory agents to increase T-cell infiltration and activity; 4) Cu_2-x_Se can serve as an effective carrier for DC-based vaccines, overcoming the limitations of conventional DC vaccines, such as low MHC-I expression and poor lymph-node accumulation, thereby enhancing immunotherapy.

#### 2.1.6 Others

Autophagy is a process by which cells degrade organelles and misfolded proteins by lysosomes, and recycle the degraded materials to provide energy for the cells (Rahdan et al., 2024; Shang et al., 2024; Zhang et al., 2025a). Autophagy plays a role in tumor progression and the efficacy of multiple cancer treatment methods, making it an important therapeutic target for cancer (Rahdan et al., 2024; Shang et al., 2024; Zhang et al., 2025a). Cu_2-x_Se was identified as a novel autophagy inhibitor that reduced the activity of lysosomal cathepsins, thereby inhibiting the degradation of autophagic contents. By inhibiting autophagy, Cu_2-x_Se significantly enhanced the anti-tumor activity of oxaliplatin, markedly suppressing tumor growth (Liu et al., 2019). Inhibiting autophagy can enhance the efficacy of multiple cancer treatment methods such as PTT, PDT, CDT, and chemotherapy (Ding et al., 2025; Liu Y. et al., 2024). The autophagy inhibitors chloroquine and hydroxychloroquine, which are currently in clinical trials, exhibit nonspecific distribution in the human body, have a short circulation half-life, and possess side effects (Liu Y. et al., 2024; Xu et al., 2022). Therefore, there is an urgent need to identify highly effective and safe autophagy inhibitors. Cu_2-x_Se is a nanoscale autophagy inhibitor, which expands the range of available autophagy inhibitors. Cu_2-x_Se can improve the efficacy of multiple cancer treatment methods such as chemotherapy, CDT, and PDT through autophagy inhibition. Because of its copper vacancies, PEG-modified Cu_2-x_Se (Cu_2-x_Se-PEG) could generate ROS under ultrasound irradiation, thereby killing tumor cells. Under ultrasonic irradiation, Cu_2-x_Se-PEG enhanced the efficiency of the Fenton-like reaction, thereby improving Cu_2-x_Se-PEG-mediated CDT (Wang X. et al., 2022). Cu_2-x_Se induced cell-cycle arrest in the G_2_/M phase and significantly suppressed the proliferation of hepatocellular carcinoma cell lines HepG2 and SMMC-7721, with IC_50_ values of 15.91 and 43.15 µM, respectively. Cu_2-x_Se impaired mitochondrial respiration by inhibiting the activity of mitochondrial electron transport chain complexes, thereby reducing adenosine triphosphate (ATP) levels, which impaired energy-dependent actin cytoskeleton assembly, inhibited the formation of tumor cell pseudopods, and consequently suppressed tumor cell migration. Cu_2-x_Se could be used to treat liver cancer by inhibiting tumor cell proliferation and migration (Liu Y. et al., 2023).

In summary, Cu_2-x_Se was a nanoscale autophagy inhibitor that might improve the efficacy of various treatment modalities, such as PTT, PDT, RT and chemotherapy, through inhibiting autophagy. Cu_2-x_Se induced cell-cycle arrest and inhibited ATP generation, suppressing tumor cell proliferation and metastasis. Although Cu_2-x_Se is a potent anticancer agent, there is currently a lack of genomic sequencing data for Cu_2-x_Se-treated cells, and the specific signaling pathways and molecular mechanisms underlying its inhibition of tumor cell proliferation and migration have not been thoroughly elucidated. This lack of information hinders the development of Cu_2-x_Se-based combination therapies with other anticancer agents.

### 2.2 Applications of Cu_2-x_Se in combination therapy

As a multifunctional nanomaterial, Cu_2-x_Se is highly suitable for combination therapy and Cu_2-x_Se-based strategies have advanced the development of combination therapeutic approaches. The applications of Cu_2-x_Se-based combination therapies are summarized in Table 2.

**TABLE 2:** Applications of Cu_2-x_Se in combination therapy.

Cu_2-x_Se- based nanosystem	Surface modification	Imaging	Treatment options	PCE	Tumor model	Administration route	Refs
Cu_2-x_Se(R837)-iRGD	RGD	-	RT + immunotherapy	-	Osteosarcoma	intravenous injection	Yao et al. (2025)
Cu_2-x_Se-DOX	-	PAI	PDT + chemotherapy	-	Glioblastoma	intravenous injection	Zhang et al. (2019)
Cu_2-x_Se/ZIF-8@Era-PEG-FA	FA	-	Chemotherapy + immunotherapy	-	Breast cancer	intravenous injection	Li K. et al. (2023)
CS−CD−Ce6	β-cyclodextrin	PAI	PDT + immunotherapy	-	Breast cancer	intravenous injection	Wang et al. (2019)
CS-P@CM	Cancer cell membrane	PAI	CDT + glutamine metabolism inhibition	-	Glioblastoma	intravenous injection	Wang L. et al. (2023)
Cu_2-x_Se@SOR@PEG	PEG	-	PTT + targeted therapy	27.6%	Hepatocellular carcinoma	intravenous injection	Huang et al. (2022)
CS@CM	Cancer cell membrane	-	PTT + catalytic therapy	37.68%	Glioblastoma	intravenous injection	Lin et al. (2025)
Cu_2-x_Se	-	-	PTT + catalytic therapy	43.7%	Breast cancer	intravenous injection	Liu J. et al. (2024)
Cu_2-x_Se@Fc	lauric acid	-	PTT + immunotherapy	70.58%	Esophageal cancer	intratumoral injection	Shi L. et al. (2025)
CS-I/J@CM	Cancer cell membrane	PAI	PTT + immunotherapy	-	Glioblastoma	intravenous injection	Wang T. et al. (2022)
Cu_2-x_Se/PDA/AIPH	-	-	PTT + chemotherapy	-	Osteosarcoma	intratumoral injection	Cheng H. et al. (2025)
DSF/Cu_2-x_Se@PCM	PCMs	PAI	PTT + chemotherapy	-	Breast cancer	intravenous injection	Pan et al. (2024)
Cu_2-x_Se-CB@MEM	Cancer cell membrane	-	PTT + chemotherapy	46.7%	Glioblastoma	intravenous injection	Song et al. (2023)
PT-V@TPDOX	FA	PAI	PTT + chemotherapy	42.8%	Breast cancer	intravenous injection	Ji et al. (2021)
Cu_2-x_Se-POED	POED	-	PTT + CDT	67.5%	Breast cancer	intravenous injection	Shen et al. (2025)
MPNPs@Cu_2-x_Se-AS1411	AS1411	PAI	PTT + CDT	82.33%	Breast cancer	intravenous injection	Liang et al. (2024)
Cu_2-x_Se-BSA	BSA	PAI	PTT + CDT	-	Breast cancer	intravenous injection	Luan et al. (2022)
CS-GOD@CM	Cancer cell membrane	PAI	PTT + CDT	62.9%	Breast cancer	intravenous injection	Wang T. et al. (2020)
ICG@Cu_2-x_Se-ZIF-8	-	-	PTT + CDT	15.5%	Breast cancer	intratumoral injection	Zou et al. (2022)
Au@Cu_2-x_Se	-	CT and PAI	PTT + CDT	56.6%	Breast cancer	intravenous injection	Zhang et al. (2021)
Ag2S-Cu(II)-Cu_2-x_Se	-	FI	PTT + CDT	61.2%	Cervical cancer	-	Han et al. (2024)
Cu_2-x_Se-Au	HS-PEG-SH	PAI and CT	PTT + RT	80.8%	Breast cancer	intravenous injection	Huang et al. (2019)
Cu_2-x_Se/Au-TAT	TAT	-	PTT + RT	64.6%	Breast cancer	intratumoral injection	Ran et al. (2023)
Cu_2-x_Se	-	-	PTT + catalytic therapy + TECT	67.0%	Colorectal cancer	intravenous injection	Yang et al. (2024)
C&P-DOX	-	-	PTT + chemotherapy + immunotherapy	-	Breast cancer	intravenous injection	Chen et al. (2025)
DOX/Mn-Cu_2-x_Se@AC	AC	-	PTT + CDT + chemotherapy	57%	Hepatocellular carcinoma	oral administration	Yin et al. (2025)
Au@Cu_2-x_Se	-	PAI	chemotherapy + RT + immunotherapy	-	Glioblastoma	intravenous injection	Zhang H. et al. (2024)
Cu_2-x_Se/Bi2Se3@PEG	PEG	CT and T1-MRI	PTT + CDT + RT	79.72%	Breast cancer	intravenous injection	Chen X. et al. (2022)
MCSN-SNO/DOX	-	-	Gas Therapy + chemotherapy + PTT	-	Hepatocellular carcinoma	intravenous injection	Huang et al. (2021)
Cu_2-x_Se@cMOF	-	-	Gas Therapy + SDT + immunotherapy	-	Breast cancer	intravenous injection	Zhao C. et al. (2024)
Cu_2-x_Se/Bi2Se3@PEG	PEG	CT and T1-MRI	PTT + PDT + CDT	60.4%	Hepatocellular carcinoma	intratumoral injection	Wang et al. (2021)
HMPC-D/G@PCM	Tetradecyl	-	PTT + CDT + chemotherapy	41.42%	Hepatocellular carcinoma	intravenous injection	Hu P. et al. (2022)
Au-Cu_2-x_Se@ZIF-8@P18	-	-	PTT + PDT + immunotherapy	-	Melanoma	intravenous injection	Yan et al. (2024)
RGD-dpGCS	RGD	PAI and CT	PTT + CDT + PCT	71.0%	Glioblastoma	intravenous injection	Shan et al. (2022)
Cu_2-x_Se-Au	-	PAI and CT	PTT + CDT + PCT	67.2%	Breast cancer	intratumoral injection	Wang Y. et al. (2020)

#### 2.2.1 PTT + chemotherapy

Currently, many studies have explored strategies for synthesizing highly toxic CuET by complexing copper ions with low-toxicity disulfiram (DSF), thereby enabling *in situ* chemotherapy at tumor sites and improving treatment efficacy (Huang et al., 2024; Kang et al., 2023). However, most studies have primarily utilized copper-based nanoparticles loaded with DSF, which cannot achieve spatiotemporally synchronized release of sufficient Cu^2+^ and DSF for rapid and sufficient CuET formation, hindering effective chemotherapy (Pan et al., 2024). Pan et al. prepared DSF/Cu_2-x_Se@PCM (DCP) by encapsulating ultrasmall Cu_2-x_Se and DSF in a thermoresponsive biodegradable organic phase-change material (PCM) composed of lauric acid and stearic acid. Below the melting point of the PCM, the DCP remained in a solid state, preventing premature reactions between Cu_2-x_Se and DSF as well as drug leakage into the bloodstream, thereby enhancing treatment safety. Under near-infrared laser irradiation, the high temperatures induced by the Cu_2-x_Se caused the PCM to melt, thereby triggering the simultaneous burst release of Cu_2-x_Se and DSF. Due to their high defect density, small size, and high surface reactivity, ultrasmall Cu_2-x_Se particles could directly chelate DSF at their surface defect sites without prior Cu^2+^ release, thereby rapidly forming abundant highly toxic Cu_2-x_Se-ET complexes for tumor-site-specific *in situ* chemotherapy. Cu_2-x_Se-ET and CuET shared similar antitumor mechanisms, while Cu_2-x_Se-ET exhibited higher antitumor activity than CuET. DCP possessed PAI capability, enabling synergistic photothermal–chemotherapy (Pan et al., 2024). This study leveraged the high-temperature degradation property of phase-change materials to simultaneously and explosively release Cu_2-x_Se and DSF at tumor sites, thereby enabling the rapid and abundant generation of highly toxic Cu_2-x_Se-ET complexes. This strategy precisely controlled the conversion of relatively low-toxicity DSF into highly toxic complexes at tumor sites, achieving safe and highly efficient *in situ* chemotherapy and offering a new approach to precision cancer therapy. Currently, effective chemotherapeutic options for glioblastoma remain limited. Many drugs cannot effectively accumulate at tumor sites because of their limited ability to cross the BBB, hindering the advancement of glioblastoma treatment (Ambele et al., 2024; Cheng W. et al., 2025; Ferreira et al., 2024). Song et al. prepared Cu_2-x_Se-CB@MEM by loading cinobufotalin (CB) onto Cu_2-x_Se and coating the resulting nanoparticles with LN229 glioblastoma cell membranes. CB inhibited inosine monophosphate dehydrogenase 1 (IMPDH1) activity, leading to tumor cell death. Because of the LN229 glioblastoma cell membrane coating, Cu_2-x_Se-CB@MEM was capable of crossing the BBB and homologously targeting glioblastoma, thereby enabling the effective accumulation of CB and Cu_2-x_Se at the tumor sites. Cu_2-x_Se-CB@MEM enabled synergistic photothermal–chemotherapy, inhibiting tumor growth (Song et al., 2023). Chemoresistance remains a major bottleneck in tumor therapy and the development of effective strategies to reverse it is of great significance (Wang et al., 2025; Zhang et al., 2025b). Ji et al. encapsulated Cu_2-x_Se within mesoporous silica (MSN) and modified the MSN shell with folic acid (FA). They then used the MSN shell to load triphenylphosphine-modified doxorubicin (TPDOX), and utilized lauric acid, a thermoresponsive phase-change material, to modify the pores of the MSN shell, thereby preparing PT-V@TPDOX. Under 808-nm laser irradiation, PT-V@TPDOX generated heat, which melted lauric acid to trigger thermoresponsive TPDOX release and impaired P-glycoprotein (P-gp) function, thereby reducing drug efflux. TPDOX actively targeted mitochondria, reversing chemotherapy resistance. PT-V@TPDOX possessed a drug-loading capacity of 10.12% and a PCE of 42.8%, reversed chemoresistance and achieved PAI-guided combined PTT and chemotherapy, producing potent antitumor efficacy against DOX-resistant breast cancer (Ji et al., 2021). This study utilized Cu_2-x_Se composites as photothermal carriers to achieve spatiotemporally controlled drug release and enhance intracellular drug retention, offering a new approach to overcoming chemoresistance (Ji et al., 2021). Cheng et al. mixed 2,2′-azobis[2-(2-imidazolin-2-yl)propane] dihydrochloride (AIPH), dopamine hydrochloride, and Cu_2-x_Se to prepare Cu_2-x_Se/PDA/AIPH. The biodegradable PDA shell helped maintain the structural stability of AIPH under physiological conditions and prevented its premature degradation during circulation. Under near-infrared laser irradiation, the high temperatures generated by Cu_2-x_Se/PDA/AIPH triggered AIPH decomposition and ROS generation, thereby inducing ROS-mediated chemotherapy. Cu_2-x_Se/PDA/AIPH enabled combined PTT with ROS-mediated chemotherapy, effectively inhibiting tumor growth in osteosarcoma-bearing mice. PDT and RT require O_2_ to generate ROS. The hypoxic nature of the TME limits ROS production during PDT and RT. This strategy utilized the high-temperature decomposition of AIPH to generate ROS, thereby overcoming the hypoxia-imposed limitation on ROS production and providing an effective approach for ROS-based tumor therapy (Cheng H. et al., 2025).

#### 2.2.2 PTT + CDT

CDT primarily uses metal ions such as Fe^2+^, Cu^+^, and Mn^2+^ to catalyze the generation of OH from intracellular H_2_O_2_ via the Fenton reaction, thereby killing tumor cells (Meng et al., 2025; Zhang X. et al., 2025). Relatively low intratumoral H_2_O_2_ levels and limited Fenton reaction efficiency restrict the efficacy of CDT (Rao et al., 2023; Shen et al., 2023; Wang Z. et al., 2022). Wang et al. prepared CS-GOD@CM by encapsulating ultrasmall Cu_2-x_Se particles loaded with glucose oxidase (GOD) within 4T1 breast cancer cell membranes. Due to the cancer cell membrane, CS-GOD@CM could specifically target breast cancer cells while minimizing the exposure of GOD, thereby reducing adverse reactions. GOD catalyzed the conversion of glucose into gluconic acid and H_2_O_2_, overcoming the issue of insufficient endogenous H_2_O_2_. Cu^+^ in Cu_2-x_Se catalyzed the decomposition of H_2_O_2_ to form •OH, thereby facilitating CDT. CS-GOD@CM-mediated PTT enhanced Fenton reaction efficiency through temperature elevation, thereby boosting CS-GOD@CM-mediated CDT. CS-GOD@CM enabled PAI-guided PTT combined with CDT, effectively inhibiting tumor growth (Wang T. et al., 2020). This study suggested that the Cu_2-x_Se nanosystem served as an effective carrier for bioenzymes and enhanced the efficiency of CDT through photothermal heating and *in situ* H_2_O_2_ supply, thereby providing insights into efficient CDT-based cancer therapy. There are currently no effective treatments for bone metastases in breast cancer (Fan et al., 2024; Wang J. et al., 2024; Yu and Zhu, 2024). The monoclonal antibody denosumab and bisphosphonate zoledronic acid, which are used clinically to treat bone metastases, may cause osteonecrosis of the jaw and hypocalcemia (Menshawy et al., 2018; Zou et al., 2022). Therefore, there is an urgent need to develop new therapeutic agents for bone metastases. Zou et al. prepared ICG@Cu_2-x_Se-ZIF-8 by encapsulating Cu_2-x_Se within zeolitic imidazolate framework-8 (ZIF-8) and using ZIF-8 to load the photosensitizer indocyanine green (ICG). The PTT mediated by Cu_2-x_Se and ICG enhanced Cu_2-x_Se-mediated CDT. The selenium in Cu_2-x_Se could regulate selenoprotein expression, inhibit tumor cell activity and osteoclast function, suppressing bone metastasis in breast cancer. ICG@Cu_2-x_Se-ZIF-8 achieved combined PTT and CDT under laser irradiation, significantly inhibiting tumor growth, alleviating tumor-induced bone resorption, and effectively suppressing breast cancer bone metastasis (Zou et al., 2022). This study demonstrated that Cu_2-x_Se has potential for treating bone metastasis by inhibiting osteoclasts, providing a new therapeutic agent for bone metastasis (Zou et al., 2022). Liang et al. grew Cu_2-x_Se nanosheets *in situ* on the surface of mesoporous platinum nanoparticles (MPNPs) and modified the Cu_2-x_Se surface with the AS1411-NH_2_ aptamer to prepare MPNPs@Cu_2-x_Se-AS1411. Due to the localized surface plasmon resonance (LSPR) effect induced by the MPNPs and copper vacancies, the MPNPs@Cu_2-x_Se-AS1411 had a PCE of 82.33%, which was higher than that of many reported photothermal agents. Due to the presence of AS1411-NH_2_, MPNPs@Cu_2-x_Se-AS1411 could actively target breast cancer cells and efficiently accumulate at the tumor sites. The Cu^2+^ in MPNPs@Cu_2-x_Se-AS1411 catalyzed H_2_O_2_ to induce the generation of highly toxic •OH for CDT. The Cu^2+^ in MPNPs@Cu_2-x_Se-AS1411 oxidized GSH to form oxidized glutathione (GSSG), thereby reducing the clearance of •OH and enhancing CDT efficacy. MPNPs@Cu_2-x_Se-AS1411 exhibited good biocompatibility and high-spatial-resolution NIR-II PAI performance, enabling synergistic PTT–CDT treatment (Liang et al., 2024). Liang et al. mixed polyvinylpyrrolidone, selenium dioxide (SeO_2_), copper chloride dihydrate (CuCl_2_.2H_2_O), and ascorbic acid with an MPNPs solution and stirred the mixture for 48 h at room temperature, resulting in the growth of Cu_2-x_Se on the MPNPs surface. This finding suggests that Cu_2-x_Se can readily grow on the surface of functional materials without the need for complex processes or harsh reaction conditions, providing a useful method for preparing novel Cu_2-x_Se-based multifunctional composites. Zhang et al. synthesized core–shell-structured Au@Cu_2-x_Se using a simple two-step reduction method. Because of enhanced LSPR coupling between Au and Cu_2-x_Se, Au@Cu_2-x_Se had a PCE of 56.6%, exhibiting excellent photothermal performance. Cu_2-x_Se-mediated PTT enhanced the catalytic efficiency of Cu^+^-mediated Fenton-like reactions, thereby improving CDT efficacy. Au possessed X-ray attenuation capability, demonstrating CT imaging property. Au@Cu_2-x_Se achieved CT/PAI-guided combined PTT and CDT, indicating that Cu_2-x_Se-based composites could be promising multifunctional nanoplatforms for combination therapy and multimodal imaging (Zhang et al., 2021). The kidneys can clear ultrasmall nanomaterials, facilitating their rapid excretion from the body and reducing accumulation-related toxicity. However, rapid renal clearance also reduces tumor-site accumulation, leading to limited therapeutic efficacy (Luan et al., 2022). Luan et al. prepared ultrasmall Cu_2-x_Se-BSA nanoparticles using bovine serum albumin (BSA) as a template (Figure 5A). Under acidic conditions, ultrasmall Cu_2-x_Se-BSA could self-assemble into aggregates, enabling tumor lysosome targeting and prolonging retention at tumor sites. Under NIR II light excitation, the self-assembled Cu_2-x_Se-BSA exhibited enhanced PAI performance, high PCE (Figure 5B), and ROS-generating capacity, inducing lysosomal lipid peroxidation (LPO) and altering lysosomal membrane permeability and inducing tumor cell death. Cu_2-x_Se-BSA exhibited limited cytotoxicity toward normal cells, which may be partly attributed to its low accumulation in these cells. After leaving the acidic TME, Cu_2-x_Se-BSA could dissociate from its aggregated state and revert to its ultrasmall size, thereby restoring renal clearability and reducing long-term accumulation-related toxicity. Cu_2-x_Se-BSA enabled PAI- and photothermal imaging-guided combined PTT and CDT, significantly inhibiting tumor growth (Figure 5C) (Luan et al., 2022). This study utilized pH-responsive self-assembled Cu_2-x_Se-BSA to resolve the fundamental conflict between rapid clearance and efficient accumulation at tumor sites, thereby offering a new approach for achieving effective and safe cancer treatment. Currently, most nanosystems operate in an always-on mode for both diagnostic signaling and therapy, resulting in poor cancer-cell specificity and hindering precise and safe cancer theranostics (Cai et al., 2024; Han et al., 2024; Pourmasoumi et al., 2024). Han et al. prepared Ag_2_S-Cu(II)-Cu_2-x_Se by cross-linking silver sulfide quantum dots (Ag_2_S QDs) with Cu_2-x_Se. The Ag_2_S QDs exhibited NIR-II fluorescence imaging (FI) and PTT capabilities. Due to the formation of Cu^2+^ coordination, fluorescence resonance energy transfer occurred between Cu_2-x_Se and Ag_2_S QDs in Ag_2_S-Cu(II)-Cu_2-x_Se, resulting in negligible NIR-II fluorescence. Under high GSH levels and acidic TME conditions, Ag_2_S-Cu(II)-Cu_2-x_Se could decompose, restoring NIR-II fluorescence and releasing Cu^2+^, thereby enabling TME-responsive NIR-II FI and CDT. The released Cu^2+^ underwent a redox reaction with GSH to form Cu^+^ and GSSG, thereby reducing GSH-mediated ROS scavenging. Cu^2+^ catalyzed intracellular H_2_O_2_ to induce the generation of ROS for CDT. Ag_2_S-Cu(II)-Cu_2-x_Se enabled NIR-II FI-guided combined PTT and CDT, demonstrating potent antitumor efficacy *in vitro* (Han et al., 2024). The TME is characterized by a dense extracellular matrix and increased interstitial fluid pressure, which hinder the accumulation and retention of nanoparticles at the tumor region, thereby decreasing the efficacy of cancer treatment (Shen et al., 2025). Shen et al. prepared pH-sensitive Cu_2-x_Se-POED by modifying Cu_2-x_Se with poly(ethylene glycol)-ortho ester-dihydrolipoic acid (POED). In the acidic TME, Cu_2-x_Se-POED underwent hydrophilicity changes and aggregated into larger particles, thereby prolonging its retention and accumulation In the tumor region. Under 808-nm laser irradiation, the aggregated Cu_2-x_Se-POED increased ROS production and enhanced the photothermal performance. With a PCE of 67.5%, Cu_2-x_Se-POED achieved a combination of CDT and PTT, effectively inhibiting breast cancer growth (Shen et al., 2025). This study used a pH-induced aggregation strategy to increase the penetration and accumulation of nanomaterials at tumor sites, improving antitumor efficacy.

**FIGURE 5:**
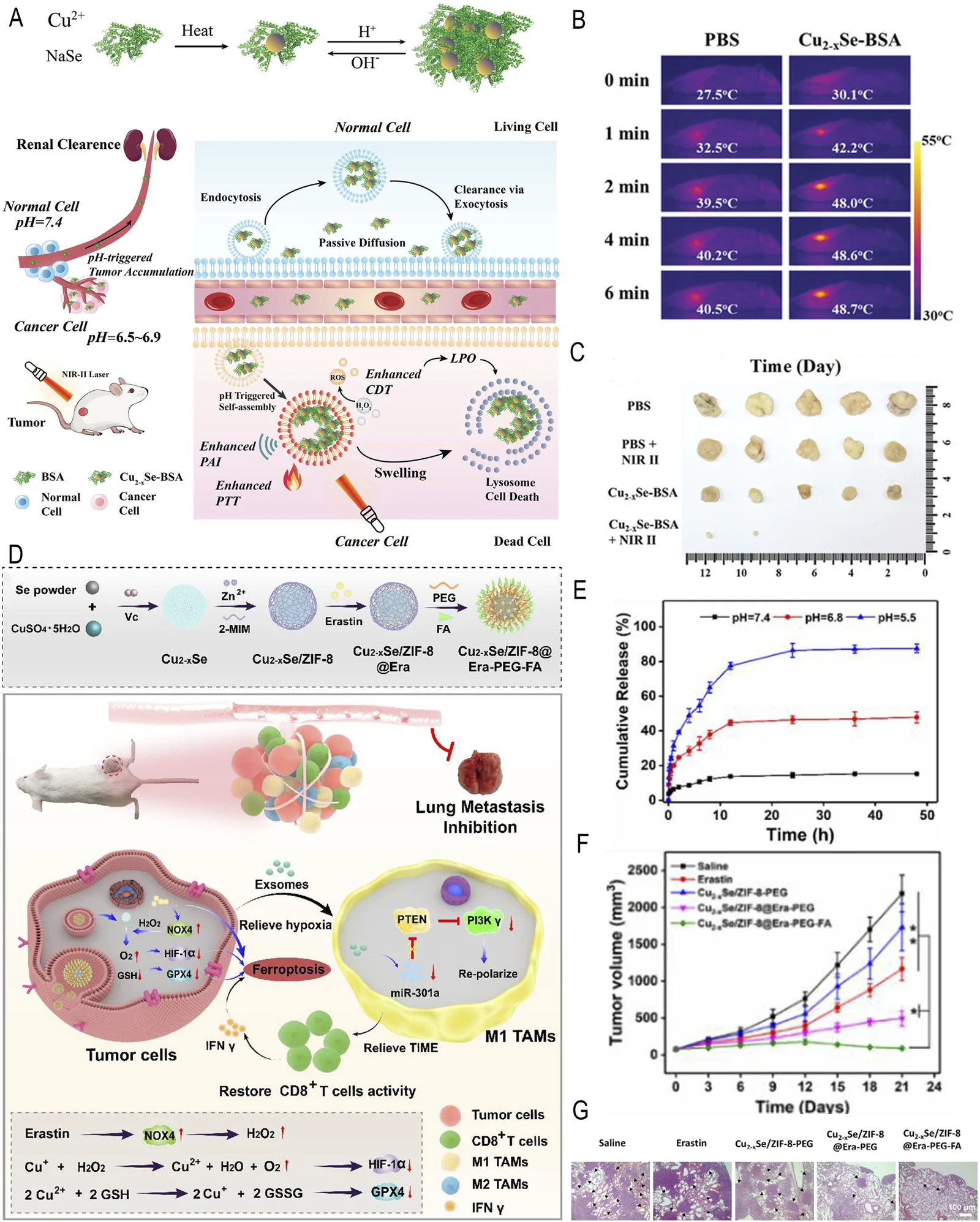
**(A)** The preparation process of Cu_2-x_Se-BSA and its application in multimodal imaging-guided photothermal–chemodynamic therapy. **(B)** Photothermal imaging of tumor-bearing mice in different groups. **(C)** Tumor tissues in different groups. Reproduced from (Luan et al., 2022). Copyright 2022 Advanced Functional Materials. **(D)** Schematic illustration of the preparation process of Cu_2-x_Se/ZIF-8@Era-PEG-FA and its use in combined ferroptosis-mediated chemotherapy and immunotherapy through O_2_ generation and GSH depletion. **(E)** pH responsive drug release behavior of Erastin. **(F)** The variations of tumor volumes in different groups. **(G)** HE staining of lung tissues in different groups. Reproduced from (Li et al., 2023a). Copyright 2023 ACS Nano.

Cu_2-x_Se-based nanoplatforms can serve as efficient platforms for integrating PTT and CDT. Cu_2-x_Se can be integrated with other photothermal agents to construct nanocomposites with high PCE, thereby enhancing photothermal performance and improving CDT efficacy by accelerating Fenton-like reaction kinetics. Cu_2-x_Se-based nanoplatforms can address major limitations of CDT by enabling *in situ* H_2_O_2_ generation and GSH depletion, thereby improving the therapeutic efficacy of CDT. Ultrasmall Cu_2-x_Se-BSA nanoparticles can self-assemble into larger aggregates in the acidic TME, thereby reducing premature renal clearance and promoting tumor accumulation. After leaving the acidic TME, these aggregates can dissociate into ultrasmall nanoparticles, enabling renal clearance and reducing long-term systemic toxicity. This strategy addressed the poor tumor accumulation of ultrasmall nanoparticles caused by premature renal clearance, thereby improving therapeutic efficacy and reducing systemic toxicity. Cu_2-x_Se-based nanoplatforms had TME-responsive NIR-II FI capability, which could enable precise tumor localization and image-guided combined CDT/PTT, thereby providing a potential platform for precision cancer theranostics.

#### 2.2.3 PTT + catalytic therapy

Nanozymes with NADH oxidase-like activity can convert NADH to NAD^+^, thereby disrupting tumor metabolism for antitumor therapy (Li D. et al., 2025). Owing to tumor heterogeneity, many NOX-like nanozymes exhibit oxygen-dependent catalytic activity under normoxic and hypoxic tumor conditions, which limits their therapeutic efficacy (Bedard et al., 2013; Liu J. et al., 2024). Owing to Cu vacancies, Cu_2-x_Se exhibited efficient NOX-like activity under both normoxic (21% O_2_) and hypoxic (2% O_2_) conditions, significantly scavenging NADH. Cu_2-x_Se also possessed GSHOx-like, POD-like, and CAT-like activities, enabling it to scavenge GSH and catalyze the conversion of H_2_O_2_ into ROS and O_2_. Leveraging its multiple nanozyme activities, Cu_2-x_Se promoted ROS generation, inhibited lactate secretion, and impaired energy production, thereby disrupting tumor metabolism to achieve catalytic therapy. Cu_2-x_Se combined PTT with catalytic therapy to effectively ablate tumor tissue (Liu J. et al., 2024). Cu_2-x_Se overcame hypoxia-induced reductions in NOX-like nanozyme activity, inhibited energy metabolism, and enhanced the efficacy of PTT (Liu J. et al., 2024). Lin et al. prepared CS@CM by encapsulating Cu_2-x_Se within glioma cell membranes. Due to the glioma cell membrane, CS@CM could effectively cross the BBB and homotypically target glioma cells. The copper ions in CS@CM catalyzed the generation of ROS from H_2_O_2_, enabling catalytic therapy. Hyperthermia generated by CS@CM-mediated PTT further enhanced its catalytic activity and amplified oxidative stress-induced damage The high temperatures generated by CS@CM-mediated PTT enhanced the catalytic performance of CS@CM and increased oxidative stress damage. By combining PTT with catalytic therapy, CS@CM efficiently inhibited the growth of orthotopic gliomas (Lin et al., 2025). However, CS@CM lacked the imaging capabilities commonly used in clinical practice, such as CT and MRI, which hindered real-time and precise evaluation of therapeutic efficacy in orthotopic gliomas.

#### 2.2.4 PDT + chemotherapy

PDT primarily involves the irradiation of a photosensitizer to generate ROS, thereby destroying tumors (Aebisher et al., 2024). PDT is a clinically used anticancer modality, showing advantages such as low toxicity and high therapeutic specificity (Zhang et al., 2024a). However, the tissue penetration of conventional PDT excitation light is limited, which severely restricts its therapeutic efficacy (Zhou et al., 2024). Zhang et al. developed Cu_2-x_Se-DOX through loading doxorubicin (DOX) onto cyclodextrin-modified ultrasmall Cu_2-x_Se nanoparticles with an average diameter of 3.3 ± 0.4 nm. Under focused ultrasound-mediated disruption of the BBB, Cu_2-x_Se-DOX could effectively cross the BBB and accumulate in glioblastoma lesions. Under 1,064 nm laser irradiation, Cu_2-x_Se could effectively handle H_2_O_2_ and O_2_ within the tumor via electron transfer and energy transfer mechanisms, generating abundant ROS to facilitate effective PDT. Cu_2-x_Se-DOX enabled PAI-guided chemotherapy combined with PDT, inhibiting the growth of orthotopic glioblastoma (Zhang et al., 2019). This study demonstrated that Cu_2-x_Se could act as an effective NIR-II photosensitizer with deep light penetration, thereby overcoming the issue of low PDT efficacy caused by shallow light penetration.

#### 2.2.5 Chemotherapy + immunotherapy

Hypoxia in the TME leads to immunosuppression and ferroptosis resistance, thereby promoting tumor progression (Feldman, 2024; Guo et al., 2024; Liu X.-Q. et al., 2024). Developing strategies to address hypoxia in the TME is of great significance (Debnath et al., 2024). Li et al. encapsulated Cu_2-x_Se within ZIF-8, loaded erastin into the ZIF-8 shell, and further modified the surface with PEG and folic acid (FA) as an active targeting ligand, thereby constructing Cu_2-x_Se/ZIF-8@Era-PEG-FA (Figure 5D). The Cu^2+^ in Cu_2-x_Se catalyzed H_2_O_2_ to induce the production of O_2_. Cu^2+^ oxidized intracellular GSH to generate Cu^+^ and GSSG. Cu_2-x_Se/ZIF-8@Era-PEG-FA enhanced intracellular O_2_ production and GSH consumption through the interconversion of Cu^+^ and Cu^2+^. This disrupted the GPX4/GSH pathway to induce ferroptosis and inhibit hypoxia-inducible factor-1α (HIF-1α) expression, thereby reducing ferroptosis resistance. Cu_2-x_Se/ZIF-8@Era-PEG-FA exhibited pH-responsive release behavior of Erastin (Figure 5E). Erastin, a ferroptosis inducer, also upregulated the expression of NOX4 protein and increased intracellular H_2_O_2_ levels, thereby enhancing Cu_2-x_Se-mediated O_2_ production. The O_2_ generated by Cu_2-x_Se/ZIF-8@Era-PEG-FA could reduce the expression of miR-301, thereby polarizing macrophages toward the M1 phenotype and increasing IFN-γ secretion by CD8^+^ T cells. These effects remodeled the immunosuppressive TME, enhanced antitumor immunity, and sensitized tumors to erastin-induced ferroptosis. Cu_2-x_Se/ZIF-8@Era-PEG-FA could actively target breast cancer cells and release erastin in a pH-responsive manner, thereby achieving a combination of ferroptosis-mediated chemotherapy and immunotherapy, and effectively inhibiting tumor growth and lung metastases (Figures 5F,G) (Li K. et al., 2023). This study demonstrated that Cu_2-x_Se could serve as an immunomodulatory nanoplatform that generated O_2_ through catalytic reactions, thereby reshaping the immunosuppressive TME, enhancing immunotherapy and sensitizing tumors to ferroptosis (Li K. et al., 2023).

#### 2.2.6 PDT + immunotherapy

Wang et al. prepared CS−CD−Ce6 by loading the photosensitizer chlorin e6 (Ce6) onto β-cyclodextrin-modified Cu_2-x_Se. CS−CD−Ce6 catalyzed the decomposition of H_2_O_2_ into O_2_ and ROS, thereby enhancing the efficacy of PDT. The abundant ROS generated by CS−CD−Ce6-mediated PDT led to tumor cell death. PDT-generated ROS induced immunogenic cell death and promoted macrophage polarization toward the M1 phenotype, thereby enhancing immunotherapy. CS−CD−Ce6 achieved PAI-guided combination of PDT and immunotherapy, effectively suppressing tumor recurrence and metastasis (Wang et al., 2019). This study demonstrated that Cu_2-x_Se not only served as an effective photosensitizer carrier but also enhanced PDT efficacy by promoting O_2_ generation and ROS production.

#### 2.2.7 CDT + glutamine metabolism inhibition

Wang et al. prepared CS-P@CM by encapsulating purpurin-loaded ultrasmall Cu_2-x_Se nanoparticles within cancer cell membranes, with purpurin serving as a glutamine metabolism inhibitor. Under focused ultrasound-mediated disruption of the BBB, CS-P@CM effectively crossed the BBB and exhibited homologous targeting toward glioblastoma cells. Purpurin inhibited glutamine metabolism, reduced intracellular GSH levels to decrease ROS scavenging, and increased intracellular H_2_O_2_ concentrations, thereby providing abundant substrates for CDT and enhancing the efficacy of Cu_2-x_Se-mediated CDT. CS-P@CM generated abundant ROS upon irradiation with NIR-II light, thereby killing tumor cells and enabling light-enhanced CDT. CS-P@CM achieved a PAI-guided combination of light-enhanced CDT and glutamine metabolism inhibition, effectively suppressing the growth of orthotopic glioblastoma tumors (Wang L. et al., 2023). Inhibiting glutamine metabolism addressed the issue of low CDT efficacy caused by elevated GSH levels and insufficient endogenous H_2_O_2_, significantly improving CDT performance (Wang L. et al., 2023).

#### 2.2.8 PTT + immunotherapy

Because glioblastoma has a complex and highly immunosuppressive TME, it is considered an immunologically “cold” tumor and has limited efficacy of immunotherapy (Wang T. et al., 2022). The immunosuppressive TME contains abundant M2-like macrophages, which promote tumor progression, as well as regulatory T cells (Tregs), which contribute to immune evasion by suppressing antigen-presenting cells and cytotoxic T lymphocytes (Li G. et al., 2025; Shi X. et al., 2025). Wang et al. utilized cancer cell membranes to encapsulate Cu_2-x_Se loaded with indoximod (IND) and the PD-L1 inhibitor JQ1, thereby preparing CS-I/J@CM. Under ultrasound irradiation, CS-I/J@CM effectively crossed the BBB and homologously targeted glioblastoma. The Cu_2-x_Se in CS-I/J@CM catalyzed the decomposition of H_2_O_2_ into O_2_ and ROS, thereby repolarizing M2-like macrophages toward the tumor-suppressive M1 phenotype and increasing M1 macrophage infiltration in the tumor region. IND in CS-I/J@CM reduced Treg infiltration at the tumor site and increased intratumoral CD8^+^ T-cell abundance, thereby reversing the tumor’s immunosuppressive microenvironment. JQ1 in CS-I/J@CM downregulated the expression of PD-L1 on tumor cells, thereby facilitating immunotherapy. Under NIR-II laser irradiation, CS-I/J@CM generated abundant ROS and induced ICD in tumor cells, improving the efficacy of immunotherapy. CS-I/J@CM achieved a PAI-guided combination of PTT and immunotherapy, effectively preventing glioblastoma recurrence (Wang T. et al., 2022). This study achieved significant immunotherapeutic effects against immunologically “cold” glioblastoma through three complementary mechanisms: remodeling the immunosuppressive TME, leading to ICD, and modulating immune checkpoint signaling, thereby providing a method for enhancing immunotherapeutic effects for “cold” tumors (Wang T. et al., 2022). Cu_2-x_Se@GG nanohydrogels loaded with the Toll-like receptor 7/8 (TLR7/8) agonist resiquimod (R848) enabled effective combined PTT and immunotherapy (Lu et al., 2023). Shi et al. prepared Cu_2-x_Se@Fc by loading the ferroptosis inducer ferrocene (Fc) onto Cu_2-x_Se and coating the nanoparticles with lauric acid, a phase-change material that melted upon heating. Under 980 nm laser irradiation, the high temperature induced by Cu_2-x_Se@Fc dissolved the lauric acid to release Fc, thereby achieving near-infrared light-responsive drug release. Cu_2-x_Se@Fc-mediated PTT and ferroptosis elicited ICD in tumor cells, improving the efficacy of anti-PD-1 blockade therapy. Cu_2-x_Se@Fc had a PCE of 70.58% and achieved synergistic photothermal–immunotherapy (Shi L. et al., 2025). The surface of Cu_2-x_Se lacks sufficient reactive functional groups and mesoporosity, which may result in limited drug-loading capacity. Researchers can utilize mesoporous materials such as metal-organic frameworks and mesoporous SiO_2_ to coat Cu_2-x_Se-based nanoparticles, thereby increasing the drug-loading capacity.

#### 2.2.9 PTT + RT

PTT can enhance tumor radiosensitivity. The combination of PTT and RT is an effective therapeutic approach in cancer treatment (Ji et al., 2022; Shi et al., 2021; Zhang et al., 2023). Huang et al. successfully synthesized dumbbell-shaped heterostructured Cu_2-x_Se-Au by utilizing the spontaneous redox reaction between Cu_2-x_Se and an HAuCl_4_ solution at room temperature. They then modified the Cu_2-x_Se-Au with dimercaptopoly(ethylene glycol) (HS-PEG-SH) to prepare CSA. ^99m^Tc-labeled CSA enabled excellent *in vivo* single-photon emission computed tomography (SPECT) imaging. Because Au is a high-atomic-number (high-Z) element, Cu_2-x_Se-Au exhibited excellent radiosensitizing effects. CSA exhibited a high photothermal conversion efficiency of 80.8%, enabling PAI- and computed tomography (CT)-guided combined PTT and radiotherapy, thereby effectively inhibiting tumor growth (Huang et al., 2019). This study suggested that nanomaterials containing high-Z elements could be combined with Cu_2-x_Se to form composites that possessed both outstanding radiosensitizing properties and high PCE, providing an efficient nanoscale platform for combined PTT and radiotherapy. Ran et al. grew AuNPs on hollow Cu_2-x_Se particles and modified the surface of the material with the cell-penetrating peptide TAT to prepare Cu_2-x_Se/Au-TAT. Cu_2-x_Se/Au-TAT effectively targeted breast cancer cells and exhibited a PCE of 64.6%, enabling highly effective combined PTT and RT (Ran et al., 2023).

#### 2.2.10 Other dual-modality combination therapies

Targeted therapy refers to the use of agents that selectively interfere with tumor-associated molecular targets, signaling pathways, or genetic alterations to inhibit tumor growth. Huang et al. modified the surface of Cu_2-x_Se with PEG and used Cu_2-x_Se to load the targeted therapeutic agent sorafenib (SOR) to prepare Cu_2-x_Se@SOR@PEG. Cu_2-x_Se@PEG had a PCE of 27.6%, indicating good photothermal effects. Cu_2-x_Se@SOR@PEG exhibited good biocompatibility, reduced the adverse effects of SOR, and achieved a combination of PTT and targeted therapy, suggesting that Cu_2-x_Se was an effective nanocarrier (Huang et al., 2022). Hypoxia in the TME can stabilize and upregulate HIF-1α, promoting tumor metastasis and chemoresistance (Basheeruddin and Qausain, 2024; Song et al., 2025). Yang et al. prepared CS-YC1@CM by encapsulating Cu_2-x_Se, loaded with the HIF-1α inhibitor YC1, within a cancer cell membrane. Yang et al. administered CS-YC1@CM intravenously via tail-vein injection and disulfiram (DSF) orally to glioblastoma-bearing mice, resulting in significant antitumor efficacy. Under ultrasound irradiation, CS-YC1@CM effectively penetrated the BBB and homologously targeted glioblastoma. The Cu^2+^ released from CS-YC1@CM combined with DSF to form toxic CuET for chemotherapy. YC1 significantly reduced HIF-1α expression, and enhanced the chemotherapeutic efficacy of CuET (Yang et al., 2022). This study combined HIF-1α suppression with CuET-mediated chemotherapy, offering a new therapeutic strategy for glioblastoma. Yao et al. prepared Cu_2-x_Se(R837)-iRGD by loading Cu_2-x_Se and imiquimod (R837) onto PLGA-PEG-iRGD. During radiotherapy, Cu_2-x_Se(R837)-iRGD generated abundant ROS and induced cuproptosis in tumor cells, thereby triggering immunogenic cell death. R837 is a Toll-like receptor 7 (TLR7) agonist that promotes dendritic cell (DC) maturation, thereby enhancing immunotherapy. The combination of Cu_2-x_Se(R837)-iRGD with anti-PD-1 antibodies achieved a synergistic effect of RT and immunotherapy, effectively inhibiting tumor growth (Yao et al., 2025). RT requires precise delineation of the target volume to avoid irradiation of normal tissues. The X-ray attenuation characteristics of osteosarcoma are similar to those of normal bone tissue, making it difficult to accurately delineate the boundary between osteosarcoma and normal bone tissue (Yao et al., 2025). This study lacked imaging studies such as fluorescence imaging (FI), CT, and MRI, which limited its ability to support precise target delineation for osteosarcoma RT.

#### 2.2.11 PTT + catalytic therapy + TECT

Thermoelectric catalytic therapy (TECT) utilizes thermoelectric catalysts to convert thermal energy into electrical energy via a temperature gradient, thereby generating ROS to destroy tumors (Wang S. et al., 2023; Yang et al., 2024). The photothermal conversion efficiency of a thermoelectric catalytic agent significantly influences temperature-gradient formation and is a key indicator of TECT efficacy (Yang et al., 2024). Currently, many thermoelectric catalytic agents have complex structures and compositions, which may make them difficult to synthesize reproducibly and pose safety concerns, restricting the application of TECT (Yang et al., 2024; Zhu Y. et al., 2024). Under 1,064 nm laser irradiation, Cu_2-x_Se exhibited strong plasmonic absorption due to abundant copper vacancies, achieving a PCE of 67% and efficient photothermal effect. Furthermore, Cu_2-x_Se exhibited POD-like and CAT-like activities, catalyzing the generation of OH and O_2_ from H_2_O_2_ for therapeutic applications. Cu_2-x_Se-mediated PTT enhanced its enzyme-like catalytic activity. The photothermal effect of Cu_2-x_Se promoted the separation of e^−^ and H^+^, leading to ROS generation and thereby successfully achieving combined PTT and TECT. Western blotting (WB) analysis showed that under 1,064 nm laser irradiation, Cu_2-x_Se promoted DLAT oligomerization, a characteristic event in cuproptosis, and reduced the levels of the iron–sulfur (Fe–S) cluster proteins FDX1 and LIAS, thereby inducing tumor cell cuproptosis. Cu_2-x_Se possessed PAI properties and suppressed tumor growth through combined cuproptosis and apoptosis. Cu_2-x_Se achieved a combination of PTT, catalytic therapy, and TECT, significantly inhibiting tumor growth (Figures 6A–E) (Yang et al., 2024). This study reported structurally and compositionally simple Cu_2-x_Se with a high PCE, providing a potentially safe and efficient thermoelectric catalytic agent for TECT.

**FIGURE 6:**
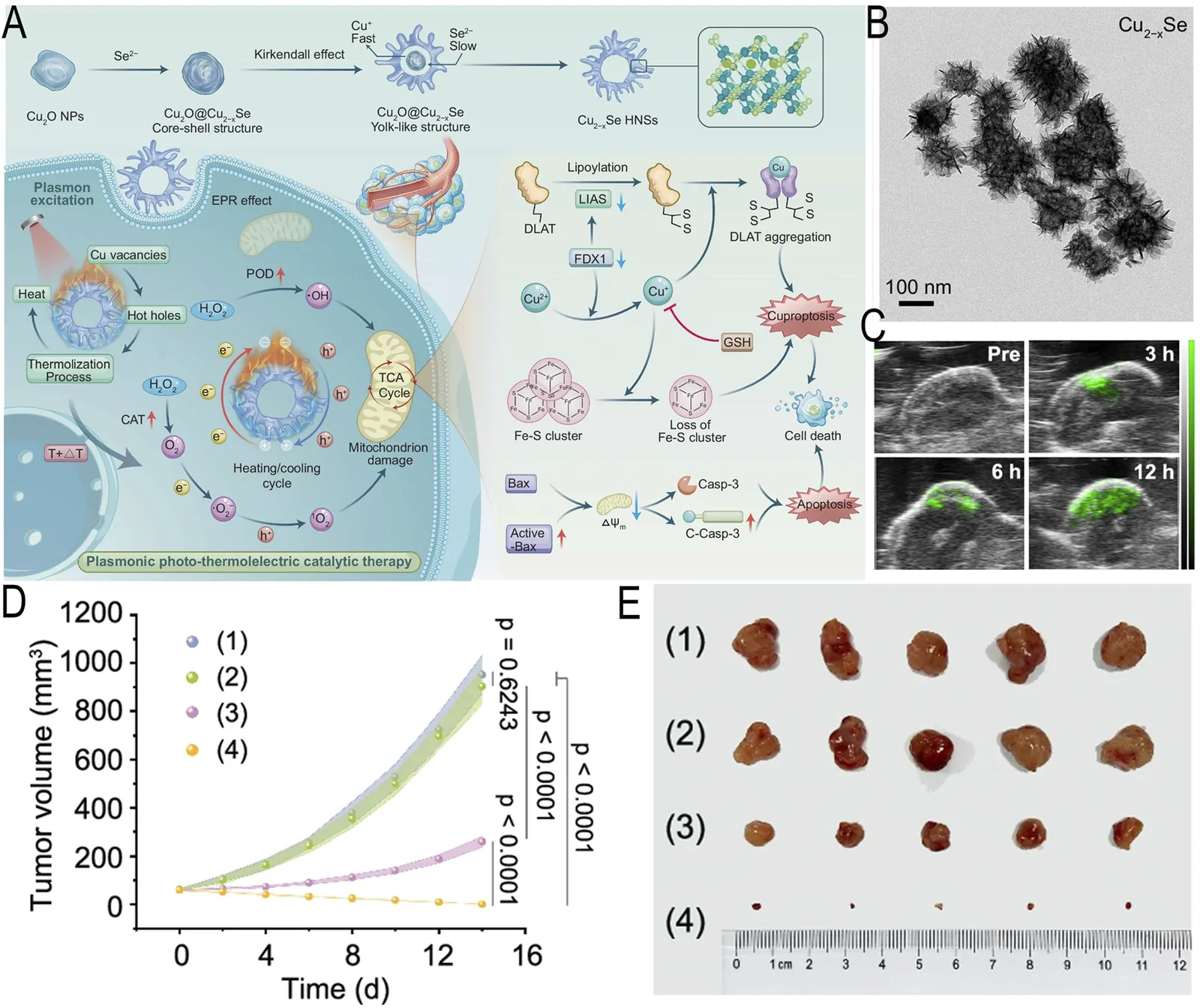
**(A)** Schematic illustration of the preparation process of urchin-like Cu_2-x_Se hollow nanospheres and their application in combined photothermal therapy, catalytic therapy, and thermoelectric catalytic therapy by the mechanisms of cuproptosis and apoptosis. **(B)** TEM images of urchin-like Cu_2-x_Se hollow nanospheres. **(C)** The photoacoustic imaging images of urchin-like Cu_2-x_Se hollow nanospheres at different time points. **(D)** Changes in tumor volume in different groups. **(E)** Images of tumor tissue in different groups. Reproduced from (Yang et al., 2024). Copyright 2024 Nature Communications.

#### 2.2.12 Chemotherapy + RT + immunotherapy

Immune checkpoint therapy (ICT) is a commonly used approach in clinical cancer immunotherapy (Wei et al., 2024). Owing to the low infiltration and reduced activity of antitumor T cells within the immunosuppressive microenvironment, ICT has shown limited efficacy in the clinical treatment of glioblastoma (Lee et al., 2025; Zhang H. et al., 2024). There is an urgent need to explore new strategies to enhance the efficacy of ICT in glioblastoma. In Au@Cu_2-x_Se (ACS), Cu_2-x_Se catalyzed H_2_O_2_ to induce the generation of O_2_ by Fenton reaction, thereby downregulating the expression of the metabolic immune checkpoint CD73. This, in turn, reduced adenosine (ADO) levels, reversed the immunosuppressive state, and enhanced the infiltration of antitumor T cells into the tumor site. Cu^2+^ released from Cu_2-x_Se reacted with the low-toxicity DSF to form the highly toxic CuET, thereby mediating chemotherapy. The Au in ACS served as a high-Z radiosensitizer, enhancing RT efficacy. The combination of RT and CuET-mediated chemotherapy induced ICD in tumor cells, promoting TAAs release, and thereby enhancing the infiltration of antitumor T-cell at the tumor site. The combination of ACS and DSF demonstrated PAI property and achieved combined chemotherapy, radiotherapy, and immunotherapy, which achieved significant antitumor effects (Figures 7A–C) (Zhang H. et al., 2024). This study simultaneously suppressed the expression of the metabolic immune checkpoint CD73 and utilized a combination of RT and chemotherapy to enhance the release of TAAs, thereby increasing the infiltration of antitumor T cells into the tumor region. This approach offered a new strategy for improving the efficacy of immunotherapy in glioblastoma. The study demonstrated that Cu_2-x_Se functioned as an immune checkpoint-modulating nanomaterial by generating O_2_ to suppress CD73 expression, thereby facilitating immunotherapy (Zhang H. et al., 2024).

**FIGURE 7:**
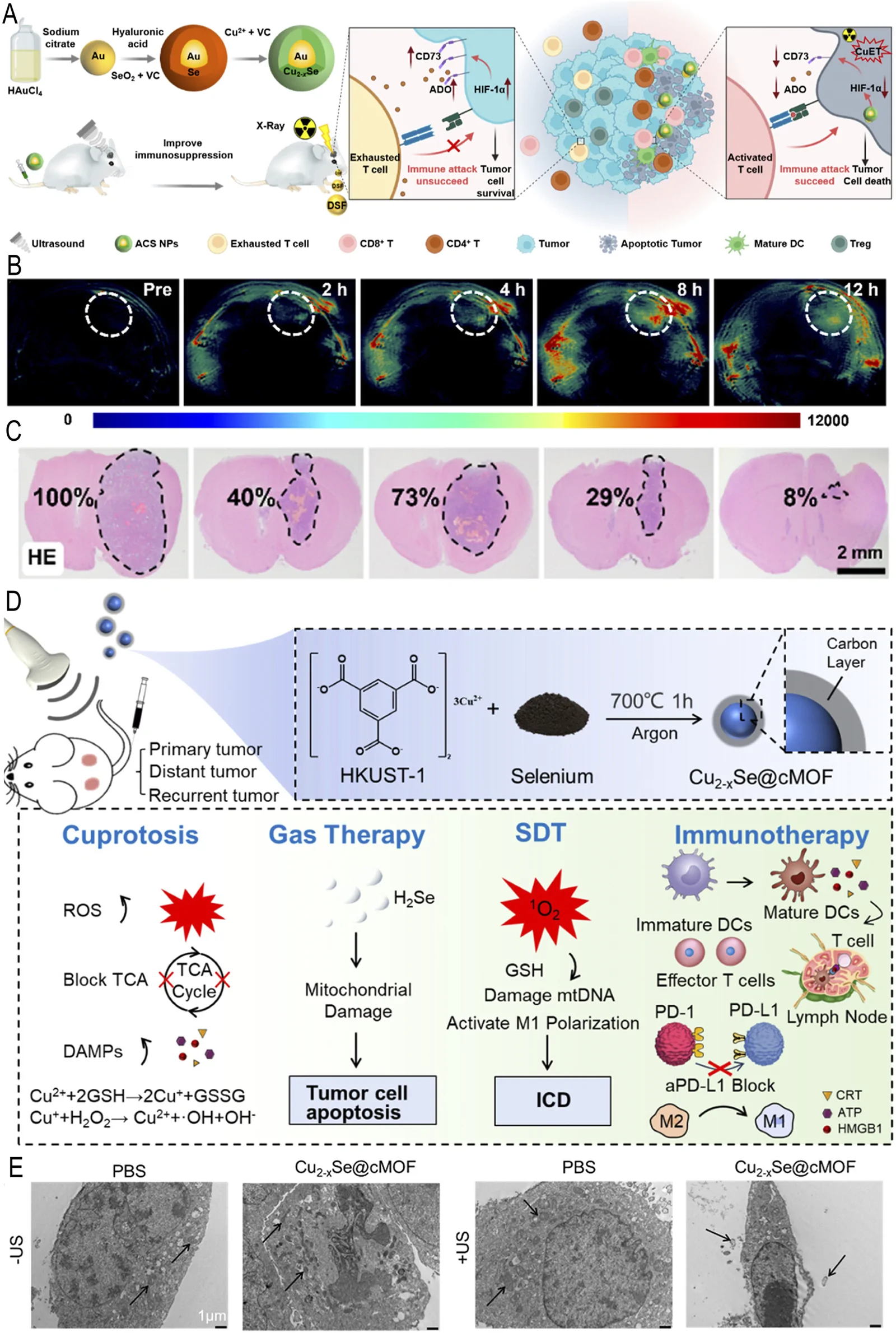
**(A)** Schematic illustration of the application of ACS in combined chemotherapy, radiotherapy, and immunotherapy. **(B)** Photoacoustic images of ACS. **(C)** HE staining images of tumor tissues in the different groups. Reproduced from (Zhang et al., 2024a). Copyright 2024 ACS Nano. **(D)** Schematic illustration of Cu_2-x_Se@cMOF for trimodal tumor therapy. **(E)** The morphology of mitochondria in different groups. Reproduced from (Zhao et al., 2024a). Copyright 2024 ACS Nano.

#### 2.2.13 Gas therapy + SDT + immunotherapy

Cuproptosis represents a distinct mode of regulated cell death, differing from apoptosis, ferroptosis, and pyroptosis. The primary mechanism of cuproptosis involves the interaction of accumulated Cu^2+^ with lipoylated proteins in the mitochondrial tricarboxylic acid cycle, leading to the aggregation of lipoylated proteins and the destabilization of Fe-S cluster proteins, triggering proteotoxic stress and inducing cell death (Li et al., 2024a; Zhu Z. et al., 2024). Cuproptosis is closely associated with tumor cell proliferation and metastasis, representing an emerging therapeutic target for tumor therapy (Hu et al., 2024; Jiang Q. et al., 2024). However, cancer therapies based on this mechanism often lack specificity and may cause adverse effects on normal tissue cells (Zhao C. et al., 2024). Zhao et al. synthesized Cu_2-x_Se@carbonized metal–organic frameworks (cMOFs) by heating a 3:1 mixture of HKUST-1 and selenium powder in a muffle furnace at 700 °C. Due to the protective effect of the carbon layer, the structure and function of Cu_2-x_Se@cMOF remained intact before entering the TME, thereby reducing toxicity to normal tissues and cells. After entering the TME, Cu_2-x_Se@cMOF released Cu^2+^ under acidic conditions and ultrasound irradiation, inducing cuproptosis in tumor cells (Figure 7E). This mechanism enabled externally triggered and tumor cell-specific cuproptosis, thereby reducing off-target toxicity associated with cuproptosis-based therapies. Under ultrasound irradiation, the selenium ions released by Cu_2-x_Se@cMOF generated H_2_Se in an acidic TME, enabling gas therapy to induce tumor cell death. Due to the presence of porphyrin-like structures, Cu_2-x_Se@cMOF generated ROS under ultrasound irradiation, enabling SDT. Under ultrasound irradiation, Cu_2-x_Se@cMOF induced ICD, enhancing the efficacy of aPD-L1-mediated immunotherapy. Cu_2-x_Se@cMOF enabled combined gas therapy, SDT, and immunotherapy, effectively integrating cuproptosis with other anticancer therapeutic modalities. This approach not only suppressed primary tumors but also effectively inhibited tumor recurrence and metastasis (Figure 7D) (Zhao C. et al., 2024). The Cu_2-x_Se@cMOF nanoplatform maintained its integrity in normal tissues but released Cu^2+^ under acidic TME conditions and ultrasound irradiation. This enabled TME-responsive and tumor cell-specific cuproptosis, providing a new approach for the safe and effective exploitation of cuproptosis in cancer therapy (Zhao C. et al., 2024).

#### 2.2.14 PTT + CDT + RT

Hypoxia and high levels of H_2_O_2_ in the TME reduce radiation-induced ROS generation, thereby promoting radioresistance (Li et al., 2024b; Yao et al., 2024; Yuan et al., 2022). Chen et al. synthesized the heterostructure Bi_2_Se_3_@Cu_2-x_Se using a simple microwave-assisted thermal injection method and applied SH-PEG-NH_2_ to modify Bi_2_Se_3_@Cu_2-x_Se to prepare Bi_2_Se_3_@Cu_2-x_Se-PEG. Compared to Bi_2_Se_3_ and Cu_2-x_Se, the heterostructure Bi_2_Se_3_@Cu_2-x_Se-PEG exhibited stronger near-infrared absorption and a higher PCE of 79.72%. Since bismuth (Bi) possessed a high X-ray attenuation coefficient, Bi_2_Se_3_@Cu_2-x_Se-PEG demonstrated radiosensitizing property and exhibited CT imaging capability with an X-ray absorption rate (38.4 HU L/g), which was higher than that of clinically used iodine-based contrast agents. Due to the paramagnetic nature of Cu^2+^, Bi_2_Se_3_@Cu_2-x_Se-PEG exhibited excellent T1-weighted MRI performance. The Cu^2+^ in Bi_2_Se_3_@Cu_2-x_Se-PEG catalyzed the generation of OH and O_2_ from high concentrations of intracellular H_2_O_2_, facilitating CDT and enhancing the efficacy of RT. Cu^2+^ scavenged GSH to generate Cu^+^, thereby disrupting tumor antioxidant defenses and enhancing the efficacy of RT. Bi_2_Se_3_@Cu_2-x_Se-PEG enabled CT and T1-weighted MRI-guided combination of PTT, CDT, and RT, achieving significant antitumor effects (Chen X. et al., 2022). This study developed a Cu_2-x_Se-based heterostructure that alleviated hypoxia and enhanced GSH depletion, providing a new strategy for enhancing the efficacy of radiotherapy and a platform for multimodal imaging-guided integrated therapy.

#### 2.2.15 Gas therapy + chemotherapy + PTT

Drug-resistant tumor cells often overexpress P-glycoprotein (P-gp), which pumps chemotherapeutic drugs out of cells and reduces intracellular drug accumulation, thereby contributing to chemoresistance (Poma et al., 2024). Huang et al. prepared MCSN-SNO/DOX by encapsulating Cu_2-x_Se in mesoporous silica (SiO_2_) and using the SiO_2_ shell to load the NO donor S-nitrosothiol (SNO) and the chemotherapeutic agent DOX. Under NIR-II laser irradiation, MCSN-SNO/DOX generated a photothermal effect that triggered NO and DOX release, achieving light-responsive and on-demand delivery of these agents. The released NO induced mitochondrial dysfunction, blocked adenosine triphosphate (ATP) synthesis, suppressed ATP-dependent P-gp expression levels, and reduced the efflux of DOX, thereby reversing chemoresistance. MCSN-SNO/DOX exhibited good biocompatibility and combined gas therapy, chemotherapy, and PTT to reverse chemoresistance, demonstrating potent antitumor effects against doxorubicin-resistant hepatocellular carcinoma cells (Huang et al., 2021).

#### 2.2.16 PTT + PDT + CDT

Wang et al. employed a cation-exchange strategy to grow Bi_2_Se_3_ on Cu_2-x_Se and used PEG to modify the resulting material, synthesizing the heterostructure Cu_2-x_Se/Bi_2_Se_3_@PEG (CB_3_@PEG). Due to the narrower bandgap of Bi_2_Se_3_, CB_3_@PEG exhibited stronger NIR absorption and a higher PCE of 60.4% compared with Cu_2-x_Se, demonstrating excellent photothermal performance. Due to the heterostructure of CB3@PEG, which resulted in stronger photo-generated electron-hole pair separation and enhanced photoredox potential, CB3@PEG generated abundant ROS under laser irradiation, leading to effective PDT. CB3@PEG could catalyze H_2_O_2_ to induce the generation of ROS, thereby facilitating CDT. Owing to the unpaired electron of Cu^2+^, Cu_2-x_Se/Bi_2_Se_3_@PEG exhibited T1-weighted MRI capability. Benefiting from the strong X-ray attenuation of bismuth, CB3@PEG demonstrated CT imaging capability. CB3@PEG achieved a photothermal imaging-, CT-, and T1-weighted MRI-guided combination of CDT, PTT, and PDT, resulting in marked suppression of tumor growth (Figures 8A,B) (Wang et al., 2021). This study demonstrated that Cu_2-x_Se-based composites could improve the photothermal performance of Cu_2-x_Se and enable multimodal imaging-guided integrated therapy, representing a valuable source of multifunctional nanoplatforms.

**FIGURE 8:**
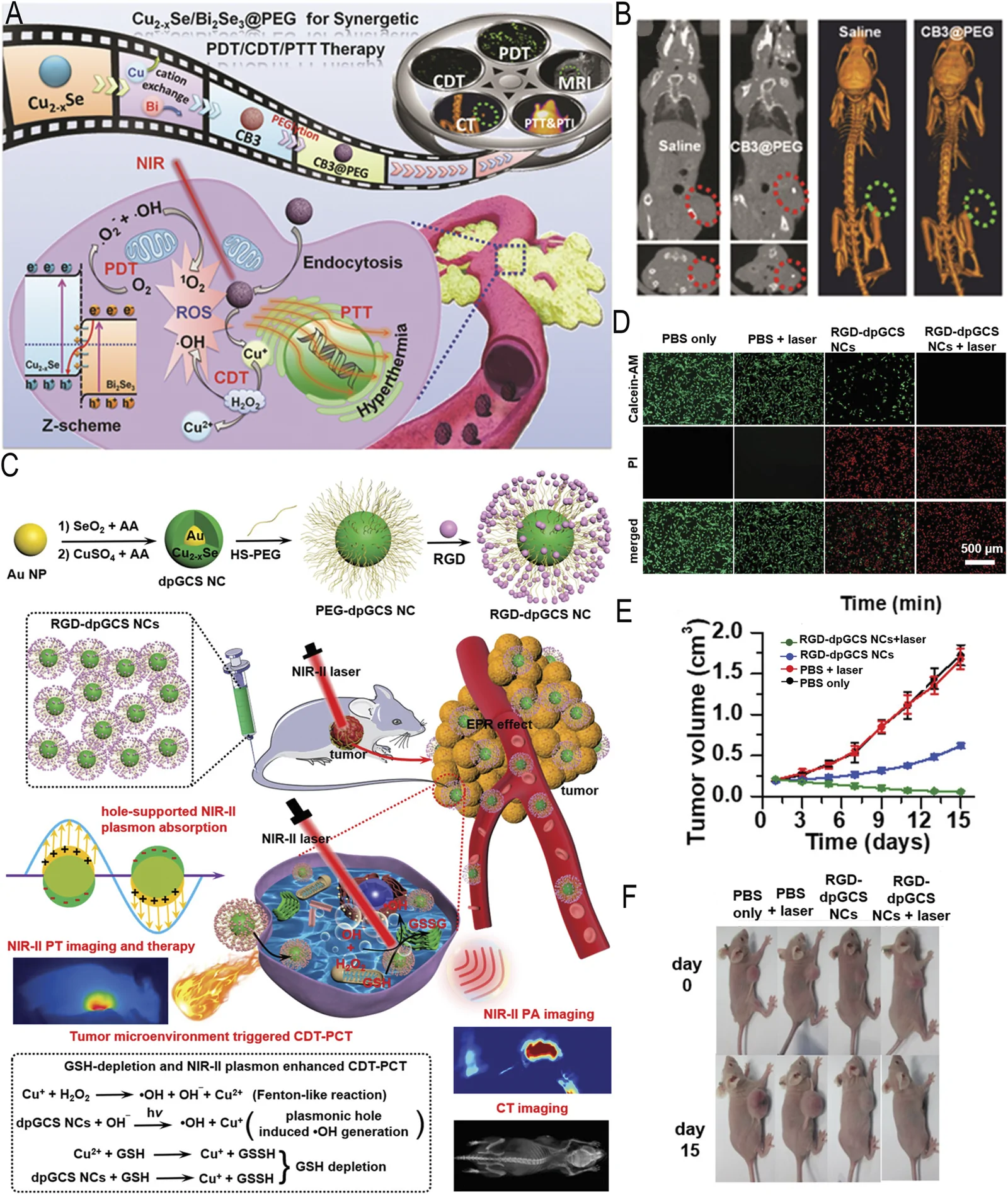
**(A)** Schematic illustration of the preparation process and application of Cu_2–x_Se/Bi2Se3@PEG. **(B)** CT images of tumor-bearing mice after Cu_2–x_Se/Bi2Se3@PEG injection. Reproduced from (Wang et al., 2021). Copyright 2021 Biomater Sci. **(C)** Schematic illustration of the synthesis process of RGD-dpGCS and its application in combination therapy. **(D)** Images of calcein-AM/PI co-stained cancer cells in different groups. **(E)** The variations of tumor volumes in different groups. **(F)** Images of tumor-bearing mice in different groups. Reproduced from (Shan et al., 2022). Copyright 2022 Small.

#### 2.2.17 PTT + CDT + chemotherapy

Hu et al. adsorbed ultrasmall Cu_2-x_Se nanoparticles onto hollow mesoporous polydopamine (HMPDA) through hydrophilic interactions, loaded DOX and GOx onto the HMPDA via π–π stacking and electrostatic interactions, and coated the surface of the prepared material with 1-tetradecanol (1-TD), resulting in the synthesis of HMPDA@Cu_2-x_Se-DOX/GOx-PCM (HMPC-D/G@PCM). Under 1,064 nm laser irradiation, PTT mediated by HMPC-D/G@PCM induced the melting of the 1-TD phase-change layer, leading to photothermally triggered release of DOX and GOx. GOx catalyzed the conversion of glucose into H_2_O_2_ and gluconic acid, compensating for the deficiency of endogenous H_2_O_2_ and supplying sufficient H_2_O_2_ substrate for CDT. Cu_2-x_Se in HMPC-D/G@PCM catalyzed the conversion of H_2_O_2_ into ROS, thereby enabling CDT. HMPC-D/G@PCM exhibited NIR-II laser- and pH-responsive drug release, thereby supporting integrated PTT, CDT, and chemotherapy, and effectively inhibiting tumor growth (Hu P. et al., 2022). This study demonstrated that Cu_2-x_Se could be adsorbed onto nanomaterials rich in reactive groups and mesopores to form composite materials, thereby overcoming the limited drug- and enzyme-loading capacity of Cu_2-x_Se caused by its lack of reactive groups and mesoporosity. Yin et al. prepared sea-urchin-like manganese-doped copper(II) selenide nanoparticles (Mn-Cu_2-x_Se) using anion exchange, loaded DOX onto Mn-Cu_2-x_Se, and coated the nanoparticles with calcium alginate/chitosan (AC) to synthesize DOX/Mn-Cu_2-x_Se@AC. Following oral administration of DOX/Mn-Cu_2-x_Se@AC, the pH-responsive swelling behavior of the AC coating helped DOX/Mn-Cu_2-x_Se overcome various biological barriers and maintained stability in the acidic gastric environment. Calcium alginate protected DOX/Mn-Cu_2-x_Se@AC from degradation in the acidic gastric environment and rapidly degraded in the mildly alkaline intestinal environment, thereby facilitating DOX/Mn-Cu_2-x_Se release for absorption in the small intestine. DOX/Mn-Cu_2-x_Se exhibited a drug-loading capacity of 0.28 mg/mg and enabled both light- and pH-responsive drug release. Mn ion doping enhanced the CDT effect of Mn-Cu_2-x_Se. Following oral administration, DOX/Mn-Cu_2-x_Se@AC effectively enabled combined chemotherapy, CDT, and PTT, significantly inhibiting tumor growth (Yin et al., 2025). This study indicated that, with appropriate surface modification, Cu_2-x_Se could serve as an oral drug-delivery carrier, thereby expanding the biomedical applications of Cu_2-x_Se (Yin et al., 2025).

#### 2.2.18 PTT + CDT + PCT

Shan et al. modified the RGD peptide onto the surface of core-shell Au@Cu_2-x_Se to prepare RGD-dpGCS. Due to the plasmonic hot-hole effect, RGD-dpGCS catalyzed the generation of •OH under NIR-II laser irradiation, thereby enabling photocatalytic therapy (PCT). RGD-dpGCS depleted GSH, thereby enhancing its CDT efficacy. RGD-dpGCS actively targeted glioblastoma and enabled PAI-, thermal imaging-, and CT-guided combined PTT, CDT, and PCT (Figure 8C-F) (Shan et al., 2022). Wang et al. synthesized a “double-faced” Cu_2-x_Se–Au structure that enabled CT-, PAI-, and infrared thermal imaging-guided combined PTT, CDT, and PCT (Wang Y. et al., 2020).

#### 2.2.19 PTT + PDT + immunotherapy

Pyroptosis is a form of programmed cell death mediated by gasdermin, which can induce ICD, promote the release of TAAs, and enhance immunotherapeutic efficacy (Ding et al., 2022; Liu Z. et al., 2024; Yan et al., 2024). Yan et al. prepared Au-Cu_2-x_Se@ZIF-8@P18 (ACS-Z-P) by encapsulating Au-Cu_2-x_Se with ZIF-8 and loading the photosensitizer P18 onto the ZIF-8 shell. Under near-infrared laser irradiation, ACS-Z-P catalyzed H_2_O_2_ decomposition to generate O_2_ and downregulated HIF-1α expression, enhancing P18-mediated PDT efficacy. ACS-Z-P enabled pH-responsive release of Zn^2+^, and the released Zn^2+^ dysregulated zinc-responsive gene/protein expression and induced DNA damage, thereby inducing pyroptosis. Cu^2+^ released from ACS-Z-P catalyzed the conversion of H_2_O_2_ into ROS, while P18-mediated PDT generated abundant ROS. Zn^2+^ and abundant ROS induced pyroptosis in tumor cells, further leading to ICD and the release of DAMPs, thereby enhancing immunotherapy. Under laser irradiation, ACS-Z-P promoted the repolarization of M2-like macrophages toward the tumor-suppressive M1 phenotype, thereby remodeling the immunosuppressive TME and enhancing immunotherapy efficacy. With a drug loading capacity of 10.13%, ACS-Z-P enabled the combination of PTT, PDT, and immunotherapy, suppressing tumor growth (Yan et al., 2024). Currently, pyroptosis-inducing nanomaterials show limited mechanistic diversity and suboptimal therapeutic efficacy (Yan et al., 2024). This study employed a combined strategy based on Zn^2+^ release and abundant ROS generation to effectively induce pyroptosis, thereby triggering ICD. Simultaneously, this strategy reshaped the immunosuppressive microenvironment and enhanced the efficacy of immunotherapy, offering a promising strategy for effectively integrating phototherapy with immunotherapy.

#### 2.2.20 PTT + chemotherapy + immunotherapy

There are few effective treatment options for TNBC. Poor drug penetration into deep tumor regions, the immunosuppressive microenvironment, and high intracellular concentrations of GSH are major obstacles to TNBC treatment (Chen et al., 2025). Chen et al. synthesized C&P by asymmetrically growing disulfide-bridged periodic mesoporous organosilica (PMO) on the surface of Cu_2-x_Se. They used C&P to load DOX, preparing C&P-DOX. Under laser irradiation, Cu_2-x_Se generated localized hyperthermia, and the asymmetric structure of C&P-DOX created a thermal gradient that drove the nanomotor into deep tumor regions. C&P-DOX functioned as a light-driven nanomotor that overcame the stromal barrier under illumination and penetrated deeply into tumor tissue, facilitating uniform DOX distribution throughout the tumor. This helped address treatment failure caused by insufficient drug distribution in certain tumor regions due to the dense extracellular matrix and abnormal vasculature of TNBC. Under the influence of high intracellular GSH concentrations, the disulfide bonds in C&P-DOX broke, inducing the degradation of PMO and enabling GSH-responsive drug release. The disulfide bonds in C&P-DOX efficiently depleted GSH, disrupted the antioxidant defense system, and enhanced the chemotherapeutic efficacy of DOX. C&P-DOX-mediated PTT and chemotherapy induced ICD, inducing the release of DAMPs and TAAs. This promoted DC maturation and enhanced T-cell activation, reversing the immunosuppressive microenvironment and thereby enhancing the immunotherapeutic efficacy of anti-PD-L1 antibodies. C&P-DOX exhibited PAI property, and enabled combined chemotherapy, PTT and immunotherapy, inhibiting breast cancer growth (Figures 9A–D) (Chen et al., 2025). This study addressed major obstacles to TNBC treatment by using light-driven nanomotors to deliver drugs into deep tumor regions, depleting GSH to mitigate chemoresistance, and combining PTT with chemotherapy to remodel the immunosuppressive TME and enhance the efficacy of immunotherapy, providing an effective strategy for treating TNBC (Chen et al., 2025).

**FIGURE 9:**
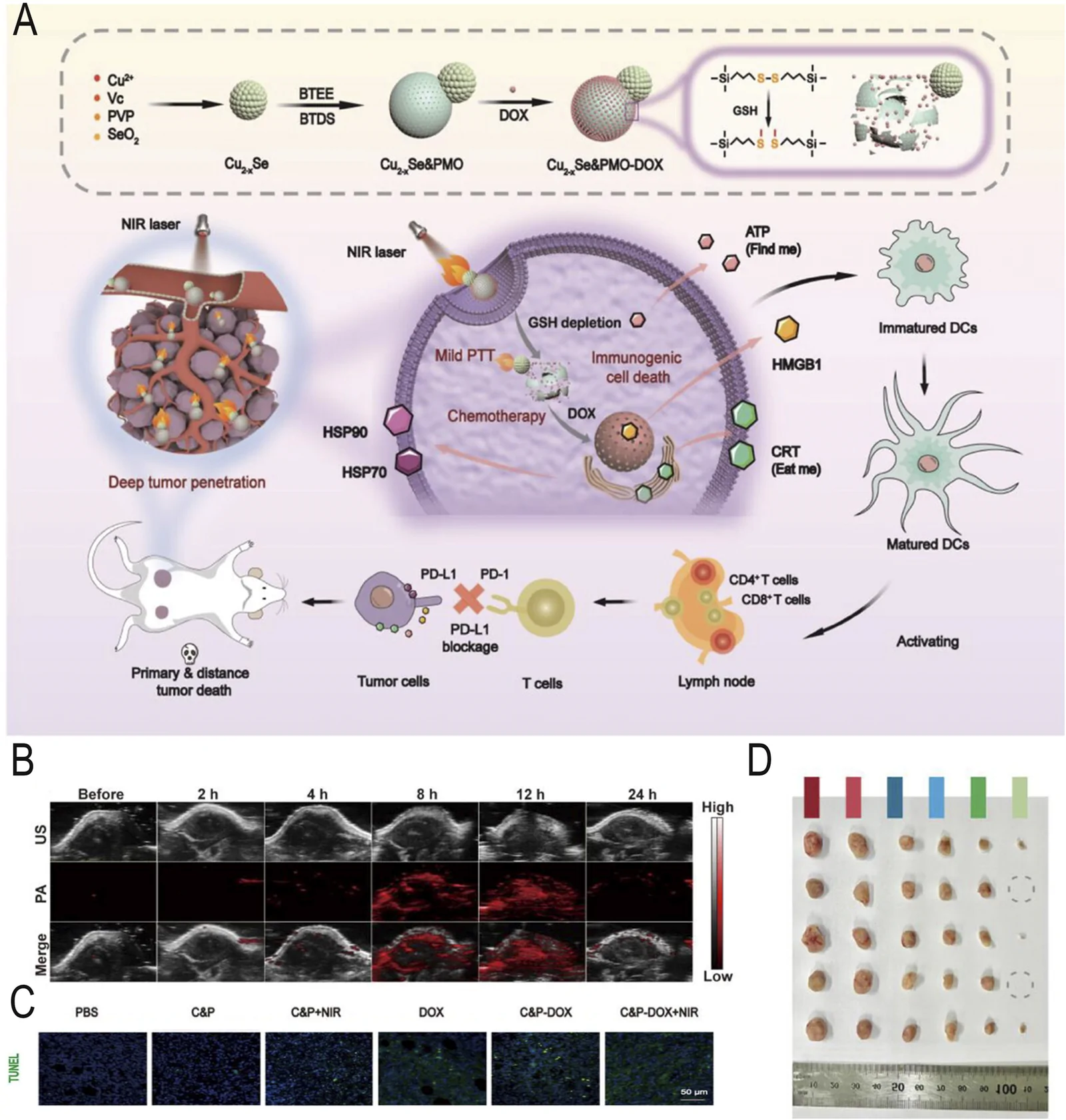
**(A)** Schematic illustration of the preparation process of C&P-DOX and its application in combined immunotherapy, chemotherapy, and photothermal therapy. **(B)** Photoacoustic imaging of C&P-DOX *in vivo*. **(C)** TUNEL staining of tumors in different groups. **(D)** Photograph of dissected tumors in different groups. Reproduced from (Chen et al., 2025). Copyright 2025 Advanced Functional Materials.

## 3 Clinical translation challenges

The clinical translation of Cu_2-x_Se remains challenging. Nanomaterials may undergo long-distance transportation and prolonged storage before being administered to patients. Because the surface of Cu_2-x_Se lacks reactive groups, it may undergo aggregation during long-term storage, which could compromise its clinical application. Additionally, Cu_2-x_Se must remain stable within the complex circulatory system to effectively reach tumor sites. Currently, various materials, including MOFs, mesoporous silica, and cell membranes, have been used to coat the surface of Cu_2-x_Se, thereby enhancing its stability in physiological environments. However, the storage stability of these Cu_2-x_Se-based nanosystems has not yet been evaluated over a 3-month period. Liposome nanosystems in clinical use demonstrate excellent aqueous dispersibility and stability. Therefore, liposomes may be used to modify Cu_2-x_Se to improve its long-term storage stability. However, liposome-modified Cu_2-x_Se has not yet been reported. Cu_2-x_Se has poor biodegradability, and its prolonged retention or repeated administration can lead to cumulative toxicity, hindering its clinical translation. The prepared ultrafine Cu_2-x_Se is easily cleared by the kidneys, thereby reducing systemic toxicity. However, ultrafine Cu_2-x_Se exhibits limited tumor accumulation at the tumor site, which reduces therapeutic efficacy. Therefore, the development of biodegradable Cu_2-x_Se is of great significance for improving its translational potential. In addition, Cu_2-x_Se may accumulate in normal tissues due to nonspecific distribution, leading to off-target toxicity and hindering its clinical application. Promoting the selective accumulation of Cu_2-x_Se at tumor sites is a key strategy for improving the efficacy of cancer treatment and reducing toxicity. From a theoretical perspective, researchers can adsorb ultra-small Cu_2-x_Se particles onto the surface of larger biodegradable nanomaterials, and further utilize cancer cell membranes or active targeting agents to modify the prepared nanocomposite. This Cu_2-x_Se-based therapeutic system can actively target tumors, leading to the efficient accumulation of Cu_2-x_Se in the tumor tissue and enhancing therapeutic efficacy. After treatment, as the larger biodegradable material degrades, the ultra-small Cu_2-x_Se particles are released from the Cu_2-x_Se-based therapeutic system and cleared by the kidneys, thereby reducing the toxic side effects caused by nonspecific distribution. However, the application of this strategy in Cu_2-x_Se-based nanomaterials has not yet been reported. The safety of nanomaterials is a key factor determining their clinical translation. Currently, safety evaluations of Cu_2-x_Se primarily involve short-term toxicity studies in tumor-bearing mice, including assessments of damage to major organs and hematological toxicity. There is a lack of research on the metabolic pathways, *in vivo* distribution, and long-term toxicity of Cu_2-x_Se in large animal models, which hinders its clinical translation. Cu_2-x_Se can be synthesized using various methods and exhibits different morphologies. Cu_2-x_Se with different particle sizes, shapes, and synthesis methods may exhibit distinct therapeutic effects and toxicity. Currently, there is a lack of comparative studies on the efficacy and toxicity of Cu_2-x_Se with different particle sizes, shapes, and synthesis methods, which hinders its clinical translation. Extensive studies are needed to identify Cu_2-x_Se formulations with superior therapeutic efficacy and reduced toxicity, thereby providing optimized candidates for further clinical translation and application. Preclinical studies and clinical applications of Cu_2-x_Se require large quantities of high-quality material. However, most current methods for synthesizing Cu_2-x_Se are limited to small-scale production, hindering its clinical translation. Therefore, there is an urgent need to develop scalable and highly reproducible synthesis methods for Cu_2-x_Se to provide sufficient material for preclinical research and future clinical applications.

## 4 Summary and future prospects

Compared with other commonly used photothermal agents such as Prussian blue (PB), copper sulfide (CuS) and gold nanorods (AuNRs), Cu_2-x_Se has its own advantages (Table 3). Cu_2-x_Se is not only a cuproptosis inducer and an autophagy inhibitor, but also a multifunctional nanomaterial that integrates imaging and various therapeutic functions. Owing to the unique advantages of Cu_2-x_Se, Cu_2-x_Se-based nanomaterials have achieved significant progress in the development of novel, potentially effective, and safe strategies for cancer treatment. Furthermore, Cu_2-x_Se-based strategies have yielded valuable findings in addressing therapeutic challenges such as tumor drug resistance, radioresistance, and immunosuppression. Cu_2-x_Se served as a promising multifunctional nanomaterial and possessed significant research value. However, Cu_2-x_Se still faces several challenges and shortcomings in tumor therapy, which must be addressed to further improve therapeutic outcomes.

**TABLE 3:** Comparative therapeutic and diagnostic profiles of Cu_2-x_Se and representative nanomaterials for tumor theranostics.

Nanomaterials	Tumor treatment performance	Photothermal stability	PCE	Catalytic activity	Imaging
Cu_2-x_Se	PTT, PDT, CDT and TECT	High	43.7% (Liu J. et al., 2024)	GSHOx-like, POD-like, NADH oxidase-like and CAT-like activities	PAI
PB	PTT and CDT	High	20.5% (Hoffman et al., 2014)	POD-like, SOD-like and CAT-like activities	MRI and PAI
CuS	PTT and CDT	High	38% (Liu et al., 2014)	GSHOx-like and POD-like activities	PAI
AuNRs	PTT and radiosensitization	Low	20.86% (Lebepe and Oluwafemi, 2022)	—	PAI and CT

Cu_2-x_Se contributes to cancer therapy by inducing cuproptosis and inhibiting autophagy. Cu_2-x_Se can arrest the cell cycle and damage mitochondria, thereby directly suppressing tumor cell proliferation and migration. Cu_2-x_Se enhanced immunotherapy through multiple mechanisms. In summary, Cu_2-x_Se demonstrates significant potential in anticancer applications. However, the specific molecular mechanisms and signaling pathways underlying the antitumor effects of Cu_2-x_Se remain unclear, which hinders the development of combination therapies that integrate Cu_2-x_Se with other therapeutic agents. Currently, there is a need to conduct genomic sequencing of Cu_2-x_Se-treated cells and perform extensive biological experiments to elucidate the specific antitumor mechanisms of Cu_2-x_Se.

Cu_2-x_Se shows CAT-like activity and catalyzes the conversion of H_2_O_2_ to O_2_ within tumors, thereby modulating the TME and alleviating immunosuppression. This enhances the efficacy of O_2_-dependent therapies such as PDT and RT, and also potentiates immunotherapy. Cu_2-x_Se possesses POD-like activity, catalyzing the conversion of H_2_O_2_ to •OH to induce efficient CDT. Adequate CAT-like and POD-like activities of Cu_2-x_Se require sufficient availability of the substrate H_2_O_2_. The concentration of intracellular H_2_O_2_ is less than 100 μM (Yu et al., 2023), limiting the efficacy of CDT. Combining Cu_2-x_Se with H_2_O_2_-generating strategies is crucial for enhancing its CDT efficacy. Currently, GOx capable of generating H_2_O_2_ has been loaded onto Cu_2-x_Se-based nanostructures to enhance CDT efficacy. However, GOx may detach from Cu_2-x_Se-based nanostructures in the complex bloodstream environment, potentially leading to adverse reactions. Cu_2-x_Se can be combined with H_2_O_2_-generating materials, such as AuNPs, ZnO_2_, MgO_2_ and CaO_2_, to form composites that address insufficient endogenous H_2_O_2_ and improve antitumor efficacy.

GSH is a main antioxidant substance that can clear ROS and reduce the efficiency of ROS-based therapeutic strategies. Copper ions released from Cu_2-x_Se can promote the oxidation of GSH to GSSG, reducing intracellular GSH levels. However, in response to GSH depletion, tumor cells may activate compensatory mechanisms that promote GSH biosynthesis, thereby restoring redox homeostasis and reducing the efficiency of tumor treatment. L-buthionine sulfoximine (BSO) can inhibit GSH biosynthesis by restricting glutamylcysteine synthetase (γ-GCS) (Jiang et al., 2026). Cu_2-x_Se-based nanoplatforms can be used to load BSO, simultaneously consuming intracellular GSH through copper ion-mediated oxidation and suppressing compensatory GSH biosynthesis via BSO. This strategy may achieve efficient intracellular GSH depletion, thereby enhancing the therapeutic efficacy of ROS-based treatment strategies.

Compared to NIR-I light, NIR-II light can penetrate deeper into tumor tissues and permits a higher maximum permissible skin exposure dose (Li N. et al., 2024). Cu_2-x_Se exhibits high photothermal stability, strong absorption in the NIR-II region, and high PCE, making it an excellent NIR-II photothermal agent. In particular, Cu_2-x_Se-based composites, such as MPNPs@Cu_2-x_Se-AS1411, Cu_2-x_Se/Bi_2_Se_3_@PEG, and Cu_2-x_Se-Au, demonstrate higher PCE and photothermal performance. Conventional PTT often results in temperatures exceeding 50 °C at the treatment site, which may cause skin damage, inflammatory responses, and even promote tumor metastasis (He X. et al., 2023). Low-temperature PTT maintains the treatment-site temperature between 42 °C and 45 °C, making it a safer therapeutic modality that is more compatible with clinical safety requirements (Wang P. et al., 2022). As the temperature of the irradiated region increases, heat shock proteins (HSPs), such as HSP70 and HSP90, are upregulated, conferring thermotolerance and limiting the efficacy of low-temperature PTT (Xin et al., 2022). However, current Cu_2-x_Se-based strategies have not fully addressed how to achieve efficient and safe low-temperature PTT, which remains a major bottleneck in PTT. Traditional Chinese medicine (gambogic acid and curcumin), small-molecule inhibitors (AT533), and glycolysis inhibitor (lonidamine) can suppress the expression of HSPs enhancing the therapeutic efficacy of low-temperature PTT (Chen et al., 2024b; Xiang et al., 2025; Zhang Z. et al., 2024, Zhang Y. et al., 2025). However, these agents are often limited by poor aqueous solubility, short circulation half-lives, and potential systemic toxicity, which hinder their clinical translation. Cu_2-x_Se-based nanoplatforms may serve as photothermal nanocarriers for these agents, addressing their limitations, improving low-temperature PTT efficacy, and facilitating their clinical application.

Cu_2-x_Se has demonstrated significant advantages in cancer treatment and holds great research value, warranting further investigation. In-depth research into Cu_2-x_Se will help overcome the challenges associated with its use in cancer treatment, facilitate the emergence of new strategies for addressing medical challenges, and promote the development of nanomedicine in cancer therapy.

## References

[B1] Aboeleneen S. B. Scully M. A. Kramarenko G. C. Day E. S. (2023). Combination cancer imaging and phototherapy mediated by membrane-wrapped nanoparticles. Int. J. Hyperth. 40, 2272066. 10.1080/02656736.2023.2272066 PMC10698846 37903544

[B2] Aebisher D. Czech S. Dynarowicz K. Misiołek M. Komosińska-Vassev K. Kawczyk-Krupka A. (2024). Photodynamic therapy: past, current, and future. Int. J. Mol. Sci. 25, 11325. 10.3390/ijms252011325 39457108 PMC11508366

[B3] Ambele M. A. Maebele L. T. Mulaudzi T. V. Kungoane T. Damane B. P. (2024). Advances in nano-delivery of phytochemicals for glioblastoma treatment. Discov. Nano 19, 216. 10.1186/s11671-024-04172-9 39718730 PMC11668727

[B4] Bao J. Wang Y. Li C. Yang C. Xu H. Liang Q. (2022). Gold-promoting-satellite to boost photothermal conversion efficiency of Cu_2-x_Se for triple-negative breast cancer targeting therapy. Mat. Today Nano 18, 100211. 10.1016/j.mtnano.2022.100211

[B5] Basheeruddin M. Qausain S. (2024). Hypoxia-inducible factor 1-Alpha (HIF-1α) and cancer: mechanisms of tumor hypoxia and therapeutic targeting. Cureus 16, e70700. 10.7759/cureus.70700 39493156 PMC11529905

[B6] Bedard P. L. Hansen A. R. Ratain M. J. Siu L. L. (2013). Tumour heterogeneity in the clinic. Nature 501, 355–364. 10.1038/nature12627 24048068 PMC5224525

[B7] Bindra A. K. Wang D. Zhao Y. (2023). Metal-organic frameworks meet polymers: from synthesis strategies to healthcare applications. Adv. Mat. 35, 2300700. 10.1002/adma.202300700 36848594

[B8] Borges F. Laureano R. S. Vanmeerbeek I. Sprooten J. Demeulenaere O. Govaerts J. (2024). Trial watch: anticancer vaccination with dendritic cells. Oncoimmunology 13, 2412876. 10.1080/2162402X.2024.2412876 39398476 PMC11469433

[B9] Cai Y. Wang W. Jiao Q. Hu T. Ren Y. Su X. (2024). Nanotechnology for the diagnosis and treatment of liver cancer. Int. J. Nanomedicine 19, 13805–13821. 10.2147/IJN.S490661 39735328 PMC11681781

[B10] Cao Y. Ding S. Hu Y. Zeng L. Zhou J. Lin L. (2024). An immunocompetent hafnium oxide-based STING nanoagonist for cancer radio-immunotherapy. Acs Nano 18, 4189–4204. 10.1021/acsnano.3c09293 38193384

[B11] Carigga Gutierrez N. M. Clainche T. L. Bulin A.-L. Leo S. Kadri M. Abdelhamid A. G. A. (2024). Engineering radiocatalytic nanoliposomes with hydrophobic gold nanoclusters for radiotherapy enhancement. Adv. Mat. 36, e2404605. 10.1002/adma.202404605 PMC11636064 39473330

[B12] Chan L. Liu Y. Chen M. Su Y. Guo J. Zhu L. (2023). Cuproptosis-driven enhancement of thermotherapy by sequentially response Cu_2-x_Se via copper chemical transition. Adv. Funct. Mat. 33, 2302054. 10.1002/adfm.202302054

[B13] Chen L. Min J. Wang F. (2022). Copper homeostasis and cuproptosis in health and disease. Signal Transduct. Target. Ther. 7, 378. 10.1038/s41392-022-01229-y 36414625 PMC9681860

[B14] Chen X. Qiu M. Liu L. Ji Q. Xu Z. Xiong Z. (2022). Intelligent Bi2Se3@Cu_2-x_Se heterostructures with enhanced photoabsorption and photoconversion efficiency for tri-modal imaging guided combinatorial cancer therapy by near-infrared II light. J. Colloid Interface Sci. 625, 614–627. 10.1016/j.jcis.2022.06.030 35764043

[B15] Chen Y. Chen Y. Wang Z. Yang L. Zhang Y. Zhang Z. (2024a). Iron-based MOF with catalase-like activity improves the synergistic therapeutic effect of PDT/ferroptosis/starvation therapy by reversing the tumor hypoxic microenvironment. J. Nanobiotechnology 22, 705. 10.1186/s12951-024-02921-7 PMC11562077 39543666

[B16] Chen Y. Ran Y. Rao Z. Wang Q. Wang J. Liu Z. (2024b). Self-assembled chemo/photothermal nanoplatform for enhanced cancer therapy: sensitizing photothermal ablation and reprogramming inflammatory microenvironment. Acs Mat. Lett. 6, 3915–3924. 10.1021/acsmaterialslett.4c01066

[B17] Chen B.-Q. Shi Y.-H. Zhang Y. Zhang D.-G. Lu X.-C. Xu Q.-X. (2025). Four-pronged nanomotor strategy achieves efficient TNBC treatment via synergistic chemo-photothermal-immune therapy. Adv. Funct. Mat. 35, 2504621. 10.1002/adfm.202504621

[B18] Cheng H. Ma B. Xia W. Yu Y. Li J. Zhang K. (2025). Synergistic photothermal and chemo-therapeutic platform utilizing Cu_2-x_Se/PDA/AIPH nanoparticles for targeted tumor eradication. Biomater. Adv. 169, 214196. 10.1016/j.bioadv.2025.214196 39854998

[B19] Cheng W. Qu H. Yang J. Chen H. Pan Y. Duan Z. (2025). Hierarchically engineered self-adaptive nanoplatform guided intuitive and precision interventions for deep-seated glioblastoma. Acs Nano 19, 557–579. 10.1021/acsnano.4c11006 39754309

[B20] Darvish L. Bahreyni Toossi M. T. Azimian H. Shakeri M. Dolat E. Ahmadizad Firouzjaei A. (2023). The role of microRNA-induced apoptosis in diverse radioresistant cancers. Cell. Signal. 104, 110580. 10.1016/j.cellsig.2022.110580 36581218

[B21] Das B. (2025). Transition metal complex-loaded nanosystems: advances in stimuli-responsive cancer therapies. Small 21, 2410338. 10.1002/smll.202410338 39663716

[B22] de Bakker T. Maes A. Dragan T. Martinive P. Penninckx S. Van Gestel D. (2024). Strategies to overcome intrinsic and acquired resistance to chemoradiotherapy in head and neck cancer. Cells 14, 18. 10.3390/cells14010018 PMC11719474 39791719

[B23] Debnath S. K. Debnath M. Ghosh A. Srivastava R. Omri A. (2024). Targeting tumor hypoxia with nanoparticle-based therapies: challenges, opportunities, and clinical implications. Pharmaceuticals 17, 1389. 10.3390/ph17101389 39459028 PMC11510357

[B24] Delgado-Corrales B. J. Chopra V. Chauhan G. (2025). Gold nanostars and nanourchins for enhanced photothermal therapy, bioimaging, and theranostics. J. Mat. Chem. B 13, 399–428. 10.1039/d4tb01420k 39575861

[B25] DeNardo D. G. Ruffell B. (2019). Macrophages as regulators of tumour immunity and immunotherapy. Nat. Rev. Immunol. 19, 369–382. 10.1038/s41577-019-0127-6 30718830 PMC7339861

[B26] Ding B. Zheng P. Tan J. Chen H. Bian Y. Yang C. (2022). Pyroptosis adjuvants: gram-scale production, Cascade catalysis, and *in situ* antitumor immunity activation. Chem. Mat. 34, 1800–1808. 10.1021/acs.chemmater.1c04048

[B27] Ding G. Liu S. Yang X. Lv H. Jia M. Li J. (2024). Metabolizable alloy clusters assemble nanoinhibitor for enhanced radiotherapy of tumor by hypoxia alleviation and intracellular PD-L1 restraint. J. Nanobiotechnology 22, 774. 10.1186/s12951-024-03057-4 39696327 PMC11657501

[B28] Ding Q. Chen H. Zhang Y. Yang J. Li M. He Q. (2025). Innovative integration of nanomedicines and phototherapy to modulate autophagy for enhanced tumor eradication. J. Control. Release Off. J. Control. Release Soc. 377, 855–879. 10.1016/j.jconrel.2024.11.071 39631701

[B29] Fan D. Li J. Li L. An M. Yang H. Zhou G. (2024). Phosphate ion-responsive and calcium peroxide-based nanomedicine for bone-targeted treatment of breast cancer bone metastasis. Adv. Healthc. Mat. 13, 2402216. 10.1002/adhm.202402216 39109966

[B30] Fecci P. E. Heimberger A. B. Sampson J. H. (2014). Immunotherapy for primary brain tumors: no longer a matter of privilege. Clin. Cancer Res. Off. J. Am. Assoc. Cancer Res. 20, 5620–5629. 10.1158/1078-0432.CCR-14-0832 25398845 PMC4235630

[B31] Feldman L. (2024). Hypoxia within the glioblastoma tumor microenvironment: a master saboteur of novel treatments. Front. Immunol. 15, 1384249. 10.3389/fimmu.2024.1384249 38994360 PMC11238147

[B32] Ferreira N. N. Leite C. M. Moreno N. S. Miranda R. R. Lins P. M. P. Rodero C. F. (2024). Nose-to-Brain delivery of biomimetic nanoparticles for glioblastoma targeted therapy. Acs Appl. Mat. Interfaces 17, 484–499. 10.1021/acsami.4c16837 PMC11783514 39692595

[B33] Fu D. Ding X. Wu J. Li C. Wang Q. Jiang J. (2018). Cationic polyelectrolyte mediated synthesis of MnO2-Based core-shell structures as activatable MRI theranostic platform for tumor cell ablation. Part. Part. Syst. Charact. 35, 1800078. 10.1002/ppsc.201800078

[B34] Guo Y. Hu P. Shi J. (2024). Nanomedicine remodels tumor microenvironment for solid tumor immunotherapy. J. Am. Chem. Soc. 146, 10217–10233. 10.1021/jacs.3c14005 38563421

[B35] Han T. Cui D. Wu M. Sun Q. Chen Y. Niu N. (2024). An effective and specific cancer theranostic nanoplatform with NIR-II fluorescence imaging-guided photothermal/chemodynamic therapy. Microchem. J. 206, 111466. 10.1016/j.microc.2024.111466

[B36] Hani U. Choudhary V. T. Ghazwani M. Alghazwani Y. Osmani R. A. M. Kulkarni G. S. (2024). Nanocarriers for delivery of anticancer drugs: current developments, challenges, and perspectives. Pharmaceutics 16, 1527. 10.3390/pharmaceutics16121527 39771506 PMC11679327

[B37] He L. Ji Q. Chi B. You S. Lu S. Yang T. (2023). Construction nanoenzymes with elaborately regulated multi-enzymatic activities for photothermal-enhanced catalytic therapy of tumor. Colloids Surf. B Biointerfaces 222, 113058. 10.1016/j.colsurfb.2022.113058 36473371

[B38] He X. Zhang S. Tian Y. Cheng W. Jing H. (2023). Research progress of nanomedicine-based mild photothermal therapy in tumor. Int. J. Nanomedicine 18, 1433–1468. 10.2147/IJN.S405020 36992822 PMC10042261

[B39] Hoffman H. A. Chakrabarti L. Dumont M. F. Sandler A. D. Fernandes R. (2014). Prussian blue nanoparticles for laser-induced photothermal therapy of tumors. Rsc Adv. 4, 29729–29734. 10.1039/c4ra05209a

[B40] Hu P. Zhao S. Shi J. Li F. Wang S. Gan Y. (2022). Precisely NIR-II-activated and pH-responsive Cascade catalytic nanoreactor for controlled drug release and self-enhanced synergetic therapy. Nanoscale 14, 12219–12231. 10.1039/d2nr00487a 35582977

[B41] Hu Y. Wang K. Ye C. (2022). “Four-in-One” nanozyme and natural enzyme symbiotic system of Cu_2-x_Se-GOx for cervical cancer therapy. Chemistry 28, e202102885. 10.1002/chem.202102885 34773414

[B42] Hu J. Zhu J. Chen T. Zhao Y. Xu Q. Wang Y. (2024). Cuproptosis in cancer therapy: mechanisms, therapeutic application and future prospects. J. Mat. Chem. B 12, 12191–12206. 10.1039/d4tb01877j 39526989

[B43] Huang R.-X. Zhou P.-K. (2020). DNA damage response signaling pathways and targets for radiotherapy sensitization in cancer. Signal Transduct. Target. Ther. 5, 60. 10.1038/s41392-020-0150-x 32355263 PMC7192953

[B44] Huang Q. Zhang S. Zhang H. Han Y. Liu H. Ren F. (2019). Boosting the radiosensitizing and photothermal performance of Cu2-xSe nanocrystals for synergetic radiophotothermal therapy of orthotopic breast cancer. ACS Nano 13, 1342–1353. 10.1021/acsnano.8b06795 30707555

[B45] Huang Y. Zhang J. Zhang Y. Shi L. Qin X. Lu B. (2021). P-glycoprotein suppression by photothermal-responsive nitric oxide releasing nanoplatform for triple-combination therapy of multidrug resistant cancer. Mat. Des. 211, 110160. 10.1016/j.matdes.2021.110160

[B46] Huang A.-T. Du J. Liu Z.-Y. Zhang G.-C. Abuduwaili W. Yan J.-Y. (2022). Sorafenib-loaded Cu_2-x_Se nanoparticles boost photothermal-synergistic targeted therapy against hepatocellular carcinoma. Nanomaterials 12, 3191. 10.3390/nano12183191 36144982 PMC9505850

[B47] Huang L. Zhu J. Wu G. Xiong W. Feng J. Yan C. (2024). A strategy of “adding fuel to the flames” enables a self-accelerating cycle of ferroptosis-cuproptosis for potent antitumor therapy. Biomaterials 311, 122701. 10.1016/j.biomaterials.2024.122701 38981152

[B48] Huang X. Hou S. Li Y. Xu G. Xia N. Duan Z. (2025). Targeting lipid metabolism via nanomedicine: a prospective strategy for cancer therapy. Biomaterials 317, 123022. 10.1016/j.biomaterials.2024.123022 39754967

[B49] Ji C. Cheng W. Hu Y. Liu Y. Liu F. Yin M. (2021). A nano vector with photothermally enhanced drug release and retention to overcome cancer multidrug resistance. Nano Today 36, 101020. 10.1016/j.nantod.2020.101020

[B50] Ji C. Zhao M. Wang C. Liu R. Zhu S. Dong X. (2022). Biocompatible tantalum nanoparticles as radiosensitizers for enhancing therapy efficacy in primary tumor and metastatic sentinel lymph nodes. ACS Nano 16, 9428–9441. 10.1021/acsnano.2c02314 35666259

[B51] Jia W. Wu Y. Xie Y. Yu M. Chen Y. (2025). Advanced polymeric nanoparticles for cancer immunotherapy: materials engineering, immunotherapeutic mechanism and clinical translation. Adv. Mat. 37, 2413603. 10.1002/adma.202413603 39797474

[B52] Jiang X. Lin W. (2025). Innate immune activation with multifunctional nanoparticles for cancer immunotherapy. Angew. Chem.-Int. Ed. 64, e202423280. 10.1002/anie.202423280 39752565

[B53] Jiang Q. Tong F. Xu Y. Liu C. Xu Q. (2024). Cuproptosis: a promising new target for breast cancer therapy. Cancer Cell Int. 24, 414. 10.1186/s12935-024-03572-2 39702350 PMC11661134

[B54] Jiang W. Huang G. Pan S. Chen X. Liu T. Yang Z. (2024). TRAIL-Driven targeting and reversing cervical cancer radioresistance by seleno-nanotherapeutics through regulating cell metabolism. Drug Resist. Updat. 72, 101033. 10.1016/j.drup.2023.101033 38157648

[B55] Jiang H. Wu H. Wang T. Jiang D. Ainiwaer T. Gu Y. (2026). Biomimetic PtPd nanozyme induce glutamylcysteine synthetase inhibition for enhanced radioimmunotherapy. Mikrochim. Acta 193, 298. 10.1007/s00604-026-08005-7 41951978

[B56] Kang X. Jadhav S. Annaji M. Huang C.-H. Amin R. Shen J. (2023). Advancing cancer therapy with copper/disulfiram nanomedicines and drug delivery systems. Pharmaceutics 15, 1567. 10.3390/pharmaceutics15061567 37376016 PMC10302862

[B57] Krayem M. Ghanem G. E. Van Gestel D. (2022). Recent advances in radiosensitivity determinants in melanoma. Curr. Opin. Oncol. 34, 131–138. 10.1097/CCO.0000000000000818 35013044

[B58] Kumari K. Singh A. Chaudhary A. Singh R. K. Shanker A. Kumar V. (2024). Neoantigen identification and dendritic cell-based vaccines for lung cancer immunotherapy. Vaccines 12, 498. 10.3390/vaccines12050498 38793749 PMC11125796

[B59] Lan Y. Li X. Liu B. Lu J. Zuo B. Wang Y. (2024). Framework nucleic acid-based nanoparticles enhance temozolomide sensitivity in glioblastoma. Drug Resist. Updat. 76, 101122. 10.1016/j.drup.2024.101122 39079407

[B60] Lebepe T. C. Oluwafemi O. S. (2022). Photothermal conversion profiling of large-scaled synthesized gold nanorods using binary surfactant with hydroquinone as a reducing agent. Nanomaterials 12, 1723. 10.3390/nano12101723 PMC9145525 35630943

[B61] Lee J. Kang Y. Lee H. Saravanakumar G. Park S. A. Ahn S. (2025). Amplifying glioblastoma immunotherapy: t cell shielding through nitric oxide/reactive oxygen species scavenging nanoparticles potentiates anti-PD-1. Biomaterials 315, 122904. 10.1016/j.biomaterials.2024.122904 39490061

[B62] Li K. Xu K. He Y. Yang Y. Tan M. Mao Y. (2023). Oxygen self-generating nanoreactor mediated ferroptosis activation and immunotherapy in triple-negative breast cancer. ACS Nano 17, 4667–4687. 10.1021/acsnano.2c10893 36861638

[B63] Li Y. Zhang Y. Dong Y. Akakuru O. U. Yao X. Yi J. (2023). Ablation of gap junction protein improves the efficiency of nanozyme-mediated Catalytic/starvation/mild-temperature photothermal therapy. Adv. Mat. 35, e2210464. 10.1002/adma.202210464 36964940

[B64] Li B. Ashrafizadeh M. Jiao T. (2024). Biomedical application of metal-organic frameworks (MOFs) in cancer therapy: stimuli-Responsive and biomimetic nanocomposites in targeted delivery, phototherapy and diagnosis. Int. J. Biol. Macromol. 260, 129391. 10.1016/j.ijbiomac.2024.129391 38242413

[B65] Li L. Pan J. Huang M. Sun J. Wang C. Xu H. (2024). Metal-phenolic networks: a promising frontier in cancer theranostics. Int. J. Nanomedicine 19, 11379–11395. 10.2147/IJN.S491421 39524920 PMC11550784

[B66] Li N. Wang Y. Li Y. Zhang C. Fang G. (2024). Recent advances in photothermal therapy at Near-Infrared-II based on 2D MXenes. Small 20, 2305645. 10.1002/smll.202305645 37775938

[B67] Li Q. Wu S. Li B. Zhou P. Wang H. Zhang X. (2024). “All-in-One” MnO2@PtAuRu nanoreactor for self-replenishing and Cascade catalytic therapy of cancer. Small 20, e2405321. 10.1002/smll.202405321 39402782

[B68] Li Y. Sun W. Yuan S. Liu X. Zhang Z. Gu R. (2024a). The role of cuproptosis in gastric cancer. Front. Immunol. 15, 1435651. 10.3389/fimmu.2024.1435651 PMC11558255 39539553

[B69] Li Y. Zhang Y. Feng N. Yu F. Liu B. (2024b). Three-in-One nanozyme for radiosensitization of bladder cancer. Int. J. Nanomedicine 19, 10873–10883. 10.2147/IJN.S463242 PMC11523926 39479176

[B70] Li D. He J. Ding G. Xin Y. Feng F. Ma S. (2025). Advancements in NADH oxidase nanozymes: bridging nanotechnology and biomedical applications. Adv. Healthc. Mat. 14, 2402785. 10.1002/adhm.202402785 39344219

[B71] Li G. Li S. Jiang Y. Chen T. An Z. (2025). Unleashing the power of immune checkpoints: a new strategy for enhancing treg cells depletion to boost antitumor immunity. Int. Immunopharmacol. 147, 113952. 10.1016/j.intimp.2024.113952 39764997

[B72] Liang J. Yang Y. Luo Y. Huang L. Zhao S. (2024). Mesoporous platinum@copper selenide-based NIR-II photothermal agents with photothermal conversion efficiency over 80% for photoacoustic imaging and targeted cancer therapy. Chem. Eng. J. 496, 154172. 10.1016/j.cej.2024.154172

[B73] Lin S. Xing H. Zeng Y. Galimova E. Chernov A. Liu G. (2025). Biomimetic Cu_2-x_Se nanoplatforms for efficient glioblastoma treatment: overcoming the blood-brain barrier and boosting Immunogenetic cell death. J. Nanobiotechnology 23, 609. 10.1186/s12951-025-03696-1 41013700 PMC12465653

[B74] Liu X. Li B. Fu F. Xu K. Zou R. Wang Q. (2014). Facile synthesis of biocompatible cysteine-coated CuS nanoparticles with high photothermal conversion efficiency for cancer therapy. Dalton Trans. 43, 11709–11715. 10.1039/c4dt00424h 24950757

[B75] Liu Y. Liu W. Huang J. Lai W. Leng F. Hu C. (2019). Cu_2-x_Se nanoparticles enhance the anticancer activity of oxaliplatin by inhibiting autophagic degradation. Nanomed 14, 1307–1321. 10.2217/nnm-2018-0284

[B76] Liu J. Yuan Y. Cheng Y. Fu D. Chen Z. Wang Y. (2022). Copper-based metal-organic framework overcomes cancer chemoresistance through systemically disrupting dynamically balanced cellular redox homeostasis. J. Am. Chem. Soc. 144, 4799–4809. 10.1021/jacs.1c11856 35192770

[B77] Liu J. Dong S. Gai S. Dong Y. Liu B. Zhao Z. (2023). Design and mechanism insight of monodispersed AuCuPt alloy nanozyme with antitumor activity. Acs Nano 17, 20402–20423. 10.1021/acsnano.3c06833 37811650

[B78] Liu Y. Liu W. Li G. Lai W. Hu C. Zhang Q. (2023). Cu_2-x_Se nanoparticles suppress cell proliferation and migration in hepatocellular carcinoma by impairing mitochondrial respiration. Biomed. Mat. 18, 035013. 10.1088/1748-605X/acc43e 36917859

[B79] Liu C. Xu X. Chen Y. Yin M. Mäkilä E. Zhou W. (2024). Metabolism-regulating nanozyme system for advanced nanocatalytic cancer therapy. Small 20, e2307794. 10.1002/smll.202307794 38168483

[B80] Liu J. Yu B. Rong M. Sun W. Lu L. (2024). A new strategy to fight tumor heterogeneity: integrating metal-defect active centers within NADH oxidase nanozymes. Nano Today 54, 102113. 10.1016/j.nantod.2023.102113

[B81] Liu X.-Q. Shi M.-Z. Bai Y.-T. Su X.-L. Liu Y.-M. Wu J.-C. (2024). Hypoxia and ferroptosis. Cell. Signal. 122, 111328. 10.1016/j.cellsig.2024.111328 39094672

[B82] Liu Y. Meng Y. Zhang J. Gu L. Shen S. Zhu Y. (2024). Pharmacology progresses and applications of chloroquine in cancer therapy. Int. J. Nanomedicine 19, 6777–6809. 10.2147/IJN.S458910 38983131 PMC11232884

[B83] Liu Z. Xu S. Chen L. Gong J. Wang M. (2024). The role of pyroptosis in cancer: key components and therapeutic potential. Cell Commun. Signal. CCS 22, 548. 10.1186/s12964-024-01932-z PMC11566483 39548573

[B84] Liu T. Lu C. Jiang X. Wang Y. Chen Z. Qi C. (2025). Nano-based strategies aiming at tumor microenvironment for improved cancer therapy. Mol. Pharm. 22, 647–677. 10.1021/acs.molpharmaceut.4c01267 39818981

[B85] Lu S. Wu Y. Liu Y. Sun X. Li J. Li J. (2023). Multifunctional photothermal hydrogel in the second near-infrared window for localized tumor therapy. ACS Appl. Bio Mat. 6, 4694–4702. 10.1021/acsabm.3c00492 37824829

[B86] Luan X. Pan Y. Zhou Y. Zhou D. Zhao W. Zeng F. (2022). Targeted self-assembly of renal clearable Cu_2-x_Se to induce lysosome swelling for multimodal imaging guided photothermal/chemodynamic synergistic therapy. Adv. Funct. Mat. 32, 2208354. 10.1002/adfm.202208354

[B87] Ma G. Wu C. (2017). Microneedle, bio-microneedle and bio-inspired microneedle: a review. J. Control. Release Off. J. Control. Release Soc. 251, 11–23. 10.1016/j.jconrel.2017.02.011 28215667

[B88] Ma M. Zhang Y. Pu K. Tang W. (2025). Nanomaterial-enabled metabolic reprogramming strategies for boosting antitumor immunity. Chem. Soc. Rev. 54, 653–714. 10.1039/d4cs00679h 39620588

[B89] Meng W. Huang L. Guo J. Xin Q. Liu J. Hu Y. (2024). Innovative nanomedicine delivery: targeting tumor microenvironment to defeat drug resistance. Pharmaceutics 16, 1549. 10.3390/pharmaceutics16121549 39771528 PMC11728492

[B90] Meng W. Chen T. Li X. Li Y. Zhang L. Xu Y. (2025). A dual-targeting biomimetic nanoplatform integrates SDT/CDT/Gas therapy to boost synergistic ferroptosis for orthotopic hepatocellular carcinoma therapy. Adv. Sci. 12, 2413833. 10.1002/advs.202413833 PMC11848614 39783849

[B91] Menshawy A. Mattar O. Abdulkarim A. Kasem S. Nasreldin N. Menshawy E. (2018). Denosumab versus bisphosphonates in patients with advanced cancers-related bone metastasis: systematic review and meta-analysis of randomized controlled trials. Support. Care Cancer 26, 1029–1038. 10.1007/s00520-018-4060-1 29387997

[B92] Pan Y.-J. Zhang Y. Chen B.-Q. Zhao Y. Wang J.-Y. Li C.-Y. (2024). NIR-II light triggered burst-release Cascade nanoreactor for precise cancer chemotherapy. Bioact. Mat. 33, 311–323. 10.1016/j.bioactmat.2023.10.033 PMC10701282 38076647

[B93] Park D. Lapteva N. Seethammagari M. Slawin K. M. Spencer D. M. (2006). An essential role for Akt1 in dendritic cell function and tumor immunotherapy. Nat. Biotechnol. 24, 1581–1590. 10.1038/nbt1262 17143278

[B94] Pi F. Deng X. Xue Q. Zheng L. Liu H. Yang F. (2023). Alleviating the hypoxic tumor microenvironment with MnO2-coated CeO2 nanoplatform for magnetic resonance imaging guided radiotherapy. J. Nanobiotechnology 21, 90. 10.1186/s12951-023-01850-1 36922836 PMC10018832

[B95] Poma P. Rigogliuso S. Labbozzetta M. Nicosia A. Costa S. Ragusa M. A. (2024). Epigenetic and cellular reprogramming of doxorubicin-resistant MCF-7 cells treated with curcumin. Int. J. Mol. Sci. 25, 13416. 10.3390/ijms252413416 39769180 PMC11679585

[B96] Porrazzo A. Cassandri M. D’Alessandro A. Morciano P. Rota R. Marampon F. (2024). DNA repair in tumor radioresistance: insights from fruit flies genetics. Cell. Oncol. 47, 717–732. 10.1007/s13402-023-00906-6 PMC12974056 38095764

[B97] Pourmasoumi P. Banihashemian S. A. Zamani F. Rasouli-Nia A. Mehrabani D. Karimi-Busheri F. (2024). Nanoparticle-based approaches in the diagnosis and treatment of brain tumors. J. Clin. Med. 13, 7449. 10.3390/jcm13237449 PMC11642644 39685907

[B98] Rahdan F. Abedi F. Dianat-Moghadam H. Sani M. Z. Taghizadeh M. Alizadeh E. (2024). Autophagy-based therapy for hepatocellular carcinoma: from standard treatments to combination therapy, oncolytic virotherapy, and targeted nanomedicines. Clin. Exp. Med. 25, 13. 10.1007/s10238-024-01527-5 PMC11611955 39621122

[B99] Ran R. Guo S. Jiang X. Qian Z. Guo Z. Wang Y. (2023). A cell-penetrating peptide modified Cu_2-x_Se/Au nanohybrid with enhanced efficacy for combined radio-photothermal therapy. Molecules 28, 423. 10.3390/molecules28010423 PMC9823383 36615627

[B100] Rao Y. Fan T. Zhou L. Fang K. Sun Y. Hu X. (2023). A positive self-amplified H2O2 and acidity circulation for boosting CDT-PTT-starvation therapy. J. Control. Release 354, 701–712. 10.1016/j.jconrel.2023.01.053 36690036

[B101] Santarsiero A. Convertini P. Iacobazzi D. Infantino V. Todisco S. (2024). Metabolic crossroad between macrophages and cancer cells: overview of hepatocellular carcinoma. Biomedicines 12, 2684. 10.3390/biomedicines12122684 39767591 PMC11727080

[B102] Saxena M. van der Burg S. H. Melief C. J. M. Bhardwaj N. (2021). Therapeutic cancer vaccines. Nat. Rev. Cancer 21, 360–378. 10.1038/s41568-021-00346-0 33907315

[B103] Shah S. Nag A. Lucke-Wold B. (2025). Autologous tumor lysate-loaded dendritic cell vaccination in glioblastoma patients: a systematic review of literature. Clin. Transl. Oncol. Off. Publ. Fed. Span. Oncol. Soc. Natl. Cancer Inst. Mex. 27, 2889–2903. 10.1007/s12094-024-03830-9 39714754

[B104] Shan B. Liu H. Li L. Lu Y. Li M. (2022). Near-infrared II plasmonic phototheranostics with glutathione depletion for multimodal imaging-guided hypoxia-tolerant chemodynamic-photocatalytic-photothermal cancer therapy triggered by a single laser. Small 18, e2105638. 10.1002/smll.202105638 34821041

[B105] Shang J. Zhao F. Xie L. Wang Y. Li B. Jin C. (2024). Trends and future directions of autophagy in osteosarcoma: a bibliometric analysis. Open Med. 19, 20241080. 10.1515/med-2024-1080 PMC11627063 39655055

[B106] Shen Y. Nie C. Pan T. Zhang W. Yang H. Ye Y. (2023). A multifunctional Cascade nanoreactor based on Fe-driven carbon nanozymes for synergistic photothermal/chemodynamic antibacterial therapy. Acta Biomater. 168, 580–592. 10.1016/j.actbio.2023.07.006 37451659

[B107] Shen X. Fang W. Sun T. Yao Y. Chen X. Xia L. (2025). pH-induced in suit aggregation of Cu_2-x_Se-POED with extended tumor retention for enhanced chemodynamic/photothermal therapy. Eur. Polym. J. 228, 113859. 10.1016/j.eurpolymj.2025.113859

[B108] Shestovskaya M. V. Luss A. L. Bezborodova O. A. Kulikov P. P. Antufrieva D. A. Plotnikova E. A. (2024). Radiosensitizing effects of heparinized magnetic iron oxide nanoparticles in colon cancer. Biomed. Pharmacother. Biomedecine Pharmacother. 175, 116668. 10.1016/j.biopha.2024.116668 38701565

[B109] Shi X. Li Q. Zhang C. Pei H. Wang G. Zhou H. (2021). Semiconducting polymer nano-radiopharmaceutical for combined radio-photothermal therapy of pancreatic tumor. J. Nanobiotechnology 19, 337. 10.1186/s12951-021-01083-0 34689758 PMC8543882

[B110] Shi Z. Hu C. Zheng X. Sun C. Li Q. (2024). Feedback loop between hypoxia and energy metabolic reprogramming aggravates the radioresistance of cancer cells. Exp. Hematol. Oncol. 13, 55. 10.1186/s40164-024-00519-1 38778409 PMC11110349

[B111] Shi L. Yu Y. Li J. Ma B. Zhang X. Yang P. (2025). NIR-Responsive Cu2 - xSe@Fc nanoparticles for photothermal-ferroptosis combination therapy in esophageal cancer. J. Nanobiotechnology 23, 356. 10.1186/s12951-025-03434-7 40380293 PMC12084917

[B112] Shi X. Askari Rizvi S. F. Yang Y. Liu G. (2025). Emerging nanomedicines for macrophage-mediated cancer therapy. Biomaterials 316, 123028. 10.1016/j.biomaterials.2024.123028 39693782

[B113] Song Z. Zhao L. Fang W. Guo S. Xu A. Zhan Z. (2023). Glioma cell membrane camouflaged cinobufotalin delivery system for combinatorial orthotopic glioblastoma therapy. Nano Res. 16, 11164–11175. 10.1007/s12274-023-5807-7

[B114] Song C. W. Kim H. Kim M.-S. Park H. J. Paek S.-H. Terezakis S. (2025). Role of HIF-1α in the responses of tumors to radiotherapy and chemotherapy. Cancer Res. Treat. 57, 1–10. 10.4143/crt.2024.255 38853541 PMC11729307

[B115] Tong P.-H. Yang J.-J. Zhou Y.-F. Tang Y.-F. Tang M.-T. Zang Y. (2025). Metal-organic frameworks (MOFs) for phototherapy and synergistic phototherapy of cancer. Coord. Chem. Rev. 526, 216381. 10.1016/j.ccr.2024.216381

[B116] Tsai C.-C. Wang C.-Y. Chang H.-H. Chang P. T. S. Chang C.-H. Chu T. Y. (2024). Diagnostics and therapy for malignant tumors. Biomedicines 12, 2659. 10.3390/biomedicines12122659 39767566 PMC11726849

[B117] Wang T. Zhang H. Han Y. Liu H. Ren F. Zeng J. (2019). Light-enhanced O2-Evolving nanoparticles boost photodynamic therapy to elicit antitumor immunity. ACS Appl. Mat. Interfaces 11, 16367–16379. 10.1021/acsami.9b03541 30994323

[B118] Wang T. Zhang H. Liu H. Yuan Q. Ren F. Han Y. (2020). Boosting H2O2-Guided chemodynamic therapy of cancer by enhancing reaction kinetics through versatile biomimetic fenton nanocatalysts and the second near-infrared light irradiation. Adv. Funct. Mat. 30, 1906128. 10.1002/adfm.201906128

[B119] Wang Y. Li Z. Hu Y. Liu J. Guo M. Wei H. (2020). Photothermal conversion-coordinated Fenton-like and photocatalytic reactions of Cu_2-x_Se-Au janus nanoparticles for tri-combination antitumor therapy. Biomaterials 255, 120167. 10.1016/j.biomaterials.2020.120167 32540756

[B120] Wang Y. Wang W. Sang D. Yu K. Lin H. Qu F. (2021). Cu_2-x_Se/Bi2Se3@PEG Z-scheme heterostructure: a multimode bioimaging guided Theranostic agent with enhanced photo/chemodynamic and photothermal therapy. Biomater. Sci. 9, 4473–4483. 10.1039/d1bm00378j 34002187

[B121] Wang P. Chen B. Zhan Y. Wang L. Luo J. Xu J. (2022). Enhancing the efficiency of mild-temperature photothermal therapy for cancer assisting with various strategies. Pharmaceutics 14, 2279. 10.3390/pharmaceutics14112279 36365098 PMC9695556

[B122] Wang T. Zhang H. Qiu W. Han Y. Liu H. Li Z. (2022). Biomimetic nanoparticles directly remodel immunosuppressive microenvironment for boosting glioblastoma immunotherapy. Bioact. Mat. 16, 418–432. 10.1016/j.bioactmat.2021.12.029 PMC8965726 35386309

[B123] Wang X. Wan Z. Li C. Meng X. Zhao L. Lu J. (2022). Defective Cu_2-x_Se crystals for ultrasound-enhanced CDT of tumor therapy. Ceram. Int. 48, 34564–34570. 10.1016/j.ceramint.2022.08.042

[B124] Wang Z. Shi Y. Shi Y. Zhang J. Hao R. Zhang G. (2022). Ultrasmall gold-coated mesoporous polydopamine nanoprobe to enhance chemodynamic therapy by self-supplying H2O2 and photothermal stimulation. Acs Appl. Mat. Interfaces 14, 54478–54487. 10.1021/acsami.2c14031 36448730

[B125] Wang L. Han Y. Gu Z. Han M. Hu C. Li Z. (2023). Boosting the therapy of glutamine-addiction glioblastoma by combining glutamine metabolism therapy with photo-enhanced chemodynamic therapy. Biomater. Sci. 11, 6252–6266. 10.1039/d3bm00897e 37534821

[B126] Wang R. Huang Z. Xiao Y. Huang T. Ming J. (2023). Photothermal therapy of copper incorporated nanomaterials for biomedicine. Biomater. Res. 27, 121. 10.1186/s40824-023-00461-z 38001505 PMC10675977

[B127] Wang S. Qiao Y. Liu X. Zhu S. Zheng Y. Jiang H. (2023). Reduced graphene oxides modified Bi2Te3 nanosheets for rapid photo-thermoelectric catalytic therapy of bacteria-infected wounds. Adv. Funct. Mat. 33, 2210098. 10.1002/adfm.202210098

[B128] Wang T. Zhang H. Han Y. Zheng Q. Liu H. Han M. (2023). Reversing T cell dysfunction to boost glioblastoma immunotherapy by paroxetine-mediated GRK2 inhibition and blockade of multiple checkpoints through biomimetic nanoparticles. Adv. Sci. 10, 2204961. 10.1002/advs.202204961 PMC10037995 36698265

[B129] Wang X.-M. Pan S. Chen L. Wang L. Dai Y.-T. Luo T. (2023). Biogenic copper selenide nanoparticles for near-infrared photothermal therapy application. ACS Appl. Mat. Interfaces 15, 27638–27646. 10.1021/acsami.3c03611 37262434

[B130] Wang A. Walden M. Ettlinger R. Kiessling F. Gassensmith J. J. Lammers T. (2024). Biomedical metal-organic framework materials: perspectives and challenges. Adv. Funct. Mat. 34, adfm.202308589. 10.1002/adfm.202308589 PMC7617264 39726715

[B131] Wang D. Han X. Liu H.-L. (2024). The role and research progress of tumor-associated macrophages in cervical cancer. Am. J. Cancer Res. 14, 5999–6011. 10.62347/FFXL7288 39803646 PMC11711540

[B132] Wang J. Liang S. Chen S. Ma T. Chen M. Niu C. (2024). Bacterial outer membrane vesicle-cancer cell hybrid membrane-coated nanoparticles for sonodynamic therapy in the treatment of breast cancer bone metastasis (vol 22, 328, 2024). J. Nanobiotechnology 22, 405. 10.1186/s12951-024-02649-4 38858780 PMC11165797

[B133] Wang P. Sun S. Bai G. Zhang R. Liang F. Zhang Y. (2024). Nanosized Prussian blue and its analogs for bioimaging and cancer theranostics. Acta Biomater. 176, 77–98. 10.1016/j.actbio.2023.12.047 38176673

[B134] Wang T. Han M. Han Y. Jiang Z. Zheng Q. Zhang H. (2024). Antigen self-presented personalized nanovaccines boost the immunotherapy of highly invasive and metastatic tumors. Acs Nano 18, 6333–6347. 10.1021/acsnano.3c11189 38349234

[B135] Wang X. Yang Y. Wang P. Li Q. Gao W. Sun Y. (2024). Oxygen self-supplying nanoradiosensitizer activates cGAS-STING pathway to enhance radioimmunotherapy of triple negative breast cancer. J. Control. Release Off. J. Control. Release Soc. 376, 794–805. 10.1016/j.jconrel.2024.10.049 39490535

[B136] Wang Y. Si Y. Wu J. Lu H. Wei J. Ding Q. (2024). Preoperative ultrasound-guided injection of nanocarbon for central lymph node dissection in patients with papillary thyroid carcinoma. Sci. Rep. 14, 29185. 10.1038/s41598-024-80364-8 39587201 PMC11589612

[B137] Wang Z. Dong M. Pan Y. Zhang L. Lei H. Zheng Y. (2024). Turning threat to therapy: a nanozyme-patch in surgical bed for convenient tumor vaccination by sustained *in situ* catalysis. Adv. Healthc. Mat. 13, e2304384. 10.1002/adhm.202304384 38301259

[B138] Wang X. Liu G. Pu X. Ren T. Zhang F. Shen M. (2025). Combating cisplatin-resistant lung cancer using a coiled-coil lipopeptides modified membrane fused drug delivery system. J. Control. Release 379, 45–58. 10.1016/j.jconrel.2025.01.004 39756686

[B139] Wei J. Li W. Zhang P. Guo F. Liu M. (2024). Current trends in sensitizing immune checkpoint inhibitors for cancer treatment. Mol. Cancer 23, 279. 10.1186/s12943-024-02179-5 39725966 PMC11670468

[B140] Wu M. Chen W. Chen Y. Zhang H. Liu C. Deng Z. (2018). Focused ultrasound-augmented delivery of biodegradable multifunctional nanoplatforms for imaging-guided brain tumor treatment. Adv. Sci. 5, 1700474. 10.1002/advs.201700474 29721406 PMC5908350

[B141] Xia C. Luo Z. Feng Z. Zhang Q. Xia C. (2025). Mechanism and application of copper-based nanomedicines in activating tumor immunity through oxidative stress modulation. Front. Pharmacol. 16, 1646890. 10.3389/fphar.2025.1646890 40717985 PMC12289571

[B142] Xiang C. Ding Q. Jiang T. Liu Y. Li C. Yang X. (2025). Reprogrammed glycolysis-induced augmentation of NIR-II excited photodynamic/photothermal therapy. Biomaterials 320, 123235. 10.1016/j.biomaterials.2025.123235 40056609

[B143] Xin Y. Sun Z. Liu J. Li W. Wang M. Chu Y. (2022). Nanomaterial-mediated low-temperature photothermal therapy *via* heat shock protein inhibition. Front. Bioeng. Biotechnol. 10, 1027468. 10.3389/fbioe.2022.1027468 PMC9595601 36304896

[B144] Xiong Y. Li J. Jiang X. Zhen W. Ma X. Lin W. (2025). Nitric oxide-releasing nanoscale metal-organic layer overcomes hypoxia and reactive oxygen species diffusion barriers to enhance cancer radiotherapy. Adv. Sci. 12, e2413518. 10.1002/advs.202413518 PMC11848595 39742392

[B145] Xu Q. Zhang H. Liu H. Han Y. Qiu W. Li Z. (2022). Inhibiting autophagy flux and DNA repair of tumor cells to boost radiotherapy of orthotopic glioblastoma. Biomaterials 280, 121287. 10.1016/j.biomaterials.2021.121287 34864449

[B146] Yan X. Chen C. Ren Y. Su T. Chen H. Yu D. (2024). A dual-pathway pyroptosis inducer based on Au-Cu_2-x_Se@ZIF-8 enhances tumor immunotherapy by disrupting the zinc ion homeostasis. Acta Biomater. 188, 329–343. 10.1016/j.actbio.2024.09.015 39278301

[B147] Yang S. Han Y. Bao B. Hu C. Li Z. (2022). Boosting the anti-tumor performance of disulfiram against glioblastoma by using ultrasmall nanoparticles and HIF-1? Inhibitor. Compos. Part B-Eng. 243, 110117. 10.1016/j.compositesb.2022.110117

[B148] Yang L. Zhao Z. Tian B. Yang M. Dong Y. Zhou B. (2024). A singular plasmonic-thermoelectric hollow nanostructure inducing apoptosis and cuproptosis for catalytic cancer therapy. Nat. Commun. 15, 7499. 10.1038/s41467-024-51772-1 PMC11362521 39209877

[B149] Yao Y. Xu R. Shao W. Tan J. Wang S. Chen S. (2024). A novel nanozyme to enhance radiotherapy effects by lactic acid scavenging, ROS generation, and hypoxia mitigation. Adv. Sci. 11, e2403107. 10.1002/advs.202403107 PMC11234405 38704679

[B150] Yao J. Chen P. Xie D. Chen J. Lin X. Xu Y. (2025). Copper-induced cuproptosis and immunogenic cell death synergize with radiotherapy and PD-1 blockade in osteosarcoma. Acs Appl. Mat. Interfaces 17, 46635–46650. 10.1021/acsami.5c08888 40763959

[B151] Yassin M. T. Al-Otibi F. O. Al-Sahli S. A. El-Wetidy M. S. Mohamed S. (2024). Metal oxide nanoparticles as efficient nanocarriers for targeted cancer therapy: addressing chemotherapy-induced disabilities. Cancers 16, 4234. 10.3390/cancers16244234 PMC11674168 39766133

[B152] Yin L. Jiang W. Liu S. Fu Y. Zhou L. Pei X. (2024). Efficacy and safety of nanoparticle albumin-bound paclitaxel plus carboplatin as neoadjuvant chemotherapy for stages III-IV, unresectable ovarian cancer: a single-arm, open-label, phase Ib/II study. Bmc Med. 22, 496. 10.1186/s12916-024-03697-1 PMC11520812 39468597

[B153] Yin H. Gao Y. Zhou X. Chen X. Hu Z. Zhang L. (2025). Oral multistage nanomedicine for synergistic chemo/chemodynamic/near-infrared-II photothermal cancer therapy. J. Colloid Interface Sci. 684, 244–253. 10.1016/j.jcis.2025.01.026 39793431

[B154] Yu X. Zhu L. (2024). Nanoparticles for the treatment of bone metastasis in breast cancer: recent advances and challenges. Int. J. Nanomedicine 19, 1867–1886. 10.2147/IJN.S442768 PMC10898486 38414525

[B155] Yu W. Jia F. Fu J. Chen Y. Huang Y. Jin Q. (2023). Enhanced transcutaneous chemodynamic therapy for melanoma treatment through cascaded fenton-like reactions and nitric oxide delivery. Acs Nano 17, 15713–15723. 10.1021/acsnano.3c02964 37565803

[B156] Yu X. Xu C. Sun J. Xu H. Huang H. Gan Z. (2024). Recent developments in two-dimensional molybdenum disulfide-based multimodal cancer theranostics. J. Nanobiotechnology 22, 515. 10.1186/s12951-024-02785-x PMC11351052 39198894

[B157] Yuan Z. Liu X. Ling J. Huang G. Huang J. Zhu X. (2022). In situ-transition nanozyme triggered by tumor microenvironment boosts synergistic cancer radio-/chemotherapy through disrupting redox homeostasis. Biomaterials 287, 121620. 10.1016/j.biomaterials.2022.121620 35709558

[B158] Zhang H. Wang T. Liu H. Ren F. Qiu W. Sun Q. (2019). Second near-infrared photodynamic therapy and chemotherapy of orthotopic malignant glioblastoma with ultra-small Cu_2-x_Se nanoparticles. Nanoscale 11, 7600–7608. 10.1039/c9nr01789e 30968107

[B159] Zhang L. Jiang C. Li B. Liu Z. Gu B. He S. (2021). A core-shell Au@Cu_2-x_Se heterogeneous metal nanocomposite for photoacoustic and computed tomography dual-imaging-guided photothermal boosted chemodynamic therapy. J. Nanobiotechnology 19, 410. 10.1186/s12951-021-01159-x 34876141 PMC8650506

[B160] Zhang H. Wang T. Liu H. Han Y. Zheng Q. Xu Q. (2022). Boost therapy of hepatocellular carcinoma by amplifying vicious cycle between mitochondrial oxidative stress and endoplasmic reticulum stress via biodegradable ultrasmall nanoparticles and old drug. Nano Today 46, 101601. 10.1016/j.nantod.2022.101601

[B161] Zhang Z. Lo H. Zhao X. Li W. Wu K. Zeng F. (2023). Mild photothermal/radiation therapy potentiates ferroptosis effect for ablation of breast cancer via MRI/PA imaging guided all-in-one strategy. J. Nanobiotechnology 21, 150. 10.1186/s12951-023-01910-6 37158923 PMC10169499

[B162] Zhang H. Yang L. Han M. Han Y. Jiang Z. Zheng Q. (2024). Boost infiltration and activity of T cells via inhibiting Ecto-5′-nucleotidase (CD73) immune checkpoint to enhance glioblastoma immunotherapy. Acs Nano 18, 23001–23013. 10.1021/acsnano.4c04553 39150454

[B163] Zhang W. Chen S. Bai Z. Gan M. Chen M. Zhang Y. (2024a). Photodynamic therapy for oral squamous cell carcinoma: current status, challenges, and prospects. Int. J. Nanomedicine 19, 10699–10710. 10.2147/IJN.S481901 PMC11512526 39464676

[B164] Zhang W. Li X. Feng C. Huang Z. Zhang C. Shuai X. (2024b). Photoferroptosis as a potent strategy for neuroblastoma treatment. Nano Today 59, 102498. 10.1016/j.nantod.2024.102498

[B165] Zhang Z. Yue X. Lan N. Zhang Y. Li Z. Jin F. (2024). Effective antitumor synergistic treatment with fiber-photothermal therapy and heat shock protein inhibitors. Acs Appl. Mat. Interfaces 17, 4368–4379. 10.1021/acsami.4c11734 39475183

[B166] Zhang J. Zhang J. Yang C. (2025a). Autophagy in brain tumors: molecular mechanisms, challenges, and therapeutic opportunities. J. Transl. Med. 23, 52. 10.1186/s12967-024-06063-0 PMC11727735 39806481

[B167] Zhang J. Zhou Y. Guo J. Yan M. Liu C. Du B. (2025b). Core-shell nanoparticles with sequential drug release depleting cholesterol for reverse tumor multidrug resistance. Acs Appl. Mat. Interfaces 17, 6689–6702. 10.1021/acsami.4c17858 39813326

[B168] Zhang X. Li Y. Zhang Y. Wang S. Zhao J. Wang T. (2025). Glutathione depletion-based pH-responsive injectable hydrogels for synergistic treatment of Colon tumor. Int. J. Biol. Macromol. 297, 139557. 10.1016/j.ijbiomac.2025.139557 39778829

[B169] Zhang Y. Yang L. Su M. Wang J. Wang R. Li H. (2025). Synergistic gambogic acid nanomedicine and mild photothermal therapy suppress metastatic lung cancer. Mat. Des. 256, 114320. 10.1016/j.matdes.2025.114320

[B170] Zhao C. Tang X. Chen X. Jiang Z. (2024). Multifaceted carbonized metal-organic frameworks synergize with immune checkpoint inhibitors for precision and augmented cuproptosis cancer therapy. ACS Nano 18, 17852–17868. 10.1021/acsnano.4c04022 38939981

[B171] Zhao X. Qi X. Liu D. Che X. Wu G. (2024). A novel approach for bladder cancer treatment: nanoparticles as a drug delivery system. Int. J. Nanomedicine 19, 13461–13483. 10.2147/IJN.S498729 39713223 PMC11662911

[B172] Zheng Y. Han Y. Wang T. Liu H. Sun Q. Hu S. (2022). Reprogramming tumor-associated macrophages via ROS-mediated novel mechanism of ultra-small Cu_2-x_Se nanoparticles to enhance anti-tumor immunity. Adv. Funct. Mat. 32, 2108971. 10.1002/adfm.202108971

[B173] Zhou X. Liu H. Zheng Y. Han Y. Wang T. Zhang H. (2020). Overcoming radioresistance in tumor therapy by alleviating hypoxia and using the HIF-1 inhibitor. ACS Appl. Mat. Interfaces 12, 4231–4240. 10.1021/acsami.9b18633 31912727

[B174] Zhou Y. Zou P. Chen X. Chen P. Shi M. Lang J. (2024). Overcoming barriers in photodynamic therapy harnessing nanogenerators strategies. Int. J. Biol. Sci. 20, 5673–5694. 10.7150/ijbs.100317 PMC11528466 39494340

[B175] Zhu L. Zhao Y. Liu T. Chen M. Qian W. P. Jiang B. (2022). Inhibition of NADPH Oxidase-ROS signal using hyaluronic acid nanoparticles for overcoming radioresistance in cancer therapy. ACS Nano 16, 18708–18728. 10.1021/acsnano.2c07440 PMC9764083 36256454

[B176] Zhu Y. Hao Q. Zhu H. Zhao R. Feng L. He S. (2024). Thermoelectric nanoheterojunction-mediated multiple energy conversion for enhanced cancer therapy. Acs Nano 18, 34257–34271. 10.1021/acsnano.4c12261 39630424

[B177] Zhu Z. Zhu K. Zhang J. Zhou Y. Zhang Q. (2024). Elucidating the evolving role of cuproptosis in breast cancer progression. Int. J. Biol. Sci. 20, 4872–4887. 10.7150/ijbs.98806 PMC11414396 39309446

[B178] Zou B. Xiong Z. He L. Chen T. (2022). Reversing breast cancer bone metastasis by metal organic framework-capped nanotherapeutics via suppressing osteoclastogenesis. Biomaterials 285, 121549. 10.1016/j.biomaterials.2022.121549 35567998

